# Chemical constituents from *Carica papaya* Linn. leaves as potential cytotoxic, EGFR^wt^ and aromatase (CYP19A) inhibitors; a study supported by molecular docking†

**DOI:** 10.1039/d1ra07000b

**Published:** 2022-03-23

**Authors:** Ashraf N. E. Hamed, Mohamed E. Abouelela, Ahmed E. El Zowalaty, Mohamed M. Badr, Mohamed S. A. Abdelkader

**Affiliations:** Department of Pharmacognosy, Faculty of Pharmacy, Minia University Minia 61519 Egypt; Department of Pharmacognosy, Faculty of Pharmacy, Al-Azhar University Assiut-Branch Assiut 71524 Egypt; Sahlgrenska Center for Cancer Research, Department of Surgery, Institute of Clinical Sciences, University of Gothenburg 40530 Gothenburg Sweden; Wallenberg Centre for Molecular and Translational Medicine, University of Gothenburg 40530 Gothenburg Sweden; Department of Pharmacology and Toxicology, Faculty of Pharmacy, Zagazig University 44519 Egypt; Department of Biochemistry, Faculty of Pharmacy, Menoufia University Menoufia 32511 Egypt; Department of Pharmacognosy, Faculty of Pharmacy, Sohag University Sohag 82524 Egypt m.salaheldin@pharm.sohag.edu.eg

## Abstract

The phytochemical investigation of the hydromethanolic extract of *Carica papaya* Linn. leaves (Caricaceae) resulted in the isolation and characterization of ten compounds, namely; carpaine (1), methyl gallate (2), loliolide (3), rutin (4), clitorin (5), kaempferol-3-*O*-neohesperidoside (6), isoquercetin (7), nicotiflorin (8) and isorhamnetin-3-*O*-β-d-glucopyranoside (9). The compounds 2, 3, 5–7 and 9 were isolated for the first time from the genus *Carica*. An *in vitro* breast cancer cytotoxicity study was evaluated with an MCF-7 cell line using the MTT assay. Methyl gallate and clitorin demonstrated the most potent cytotoxic activities with an IC_50_ of 1.11 ± 0.06 and 2.47 ± 0.14 μM, respectively. Moreover, methyl gallate and nicotiflorin exhibited potential EGFR^wt^ kinase inhibition activities with an IC_50_ of 37.3 ± 1.9 and 41.08 ± 2.1 nM, respectively, compared with the positive control erlotinib (IC_50_ = 35.94 ± 1.8 nM). On the other hand, clitorin and nicotiflorin displayed the strongest aromatase kinase inhibition activities with an IC_50_ of 77.41 ± 4.53 and 92.84 ± 5.44 nM, respectively. Clitorin was comparable to the efficacy of the standard drug letrozole (IC_50_ = 77.72 ± 4.55). Additionally, molecular docking simulations of the isolated compounds to EGFR and human placental aromatase cytochrome P450 (CYP19A1) were evaluated. Methyl gallate linked with the EGFR receptor through hydrogen bonding with a pose score of −4.5287 kcal mol^−1^ and RMSD value of 1.69 Å. Clitorin showed the strongest interaction with aromatase (CYP19A1) for the breast cancer receptor with a posing score of −14.2074 and RMSD value of 1.56 Å. Compounds (1–3) possessed a good bioavailability score with a 0.55 value.

## Abstract 2

The phytochemical investigation of the hydromethanolic extract of *Carica papaya* Linn. leaves (Caricaceae) resulted in the isolation and characterization of ten compounds.

## Graphical abstract

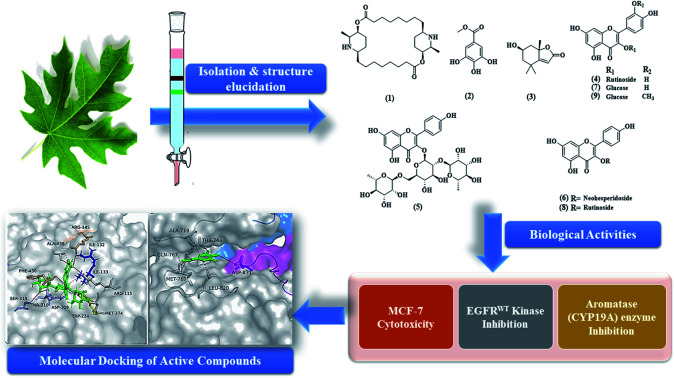

## 1. Introduction

Pawpaw or Papaya (*Carica papaya* Linn.) is an evergreen tree that belongs to the family Caricaceae, it is indigenous to Central America and the South of Mexico. It is commonly grown in the subtropical and tropical regions and cultivated in many countries worldwide.^1,2^*C. papaya* is considered the most valuable species in the family due to its nutritional and therapeutic benefits.^3^ Its leaves are traditionally used as a cardiotonic, vermifuge, febrifuge and as a treatment for colic, dengue fever, beriberi, asthma, cancer and stomach troubles in India and Australia.^4,5^ In addition, the different parts of the Pawpaw plant have been used for their therapeutic applications and strong activities as an antibacterial, antiviral, antitumor, antidiabetic, anti-inflammatory and management of neurodegeneration.^6^ Numerous scientific studies have confirmed that *C. papaya* contains alkaloids, glycosides, tannins, saponins, flavonoids and glycosides which may be responsible for its therapeutic activity.^5^

Cancer is a major cause of mortality with a higher incidence in developed and developing countries. Worldwide, about 19.3 million new cancer cases and an estimated 10.0 million deaths due to cancer were reported in 2020. Female breast cancer was the most commonly diagnosed cancer, with an estimated 11.7% of new cases.^7^ In Egypt, breast cancer incidence accounts for about 38.85% of total diagnosed female cancer cases.^8^

Despite the advances in cancer research and clinical trials of promising new therapies, there is still a great demand for the discovery of new safe and effective drugs with low adverse effects on human health.^9^ Natural products have a strong role in the development of anti-cancer agents, thus various drug discovery programs continue to invest in this outstanding source.^10^

Many scientific studies have reported the effect of *C. papaya* leaves extract on the treatment of breast cancer, cervical carcinoma, hepatocellular carcinoma, osteosarcoma, lung adenocarcinoma and many other types of cancer.^6,11^

The EGFR tyrosine kinase is critical for hormone receptor positive breast cancer and upregulation of EGFR leads to aberrant signalling.^12^ It has been reported that 57% of breast carcinomas express EGFR^wt^.^13^ In addition, triple negative breast cancer (TNBC) characterized by low expression of estrogen, progesterone and Her2 receptors, is associated with overexpression of EGFR.^14^ In addition to the role of EGFR in breast cancer progression, aromatase enzyme is critical for breast cancer development and progression. Aromatase catalyzes the final rate-limiting step in estrogen biosynthesis. Aromatase catalyzes a three-step reaction on androgen substrates. The third step of the reaction leads to the aromatization of the A-ring. Aromatase is also highly expressed in breast cancer producing higher levels of estrogen.^15^ However, breast cancer cells constantly develop resistance to aromatase inhibitors by acquiring estrogen receptor mutations, truncation and upregulation of ER-related transcription factors activator protein 1 (AP1) and NF-κB, aromatase inhibitors are effective in breast cancer treatment.^16–18^

Even though previous studies on the effect of *C. papaya* and its leaves on breast cancer, the effect of its bioactive compounds and possible mechanism of action on specific cancer targets still needs more exploration. This provoked us to carry out an extensive phytochemical study of *C. papaya* Linn. leaves to isolate the active metabolites and test their effect on the MCF-7 breast cancer cell line as well as evaluation of their epithelial growth factor receptor (EGFR^wt^) kinase and aromatase (CYP19A) enzyme inhibition activity. In addition, the explanation of the potent compounds possible binding mode to their targets by *in silico* molecular docking studies.

## 2. Result and discussion

### 2.1. Structure elucidation of the isolated compounds

**Fig. 1:**
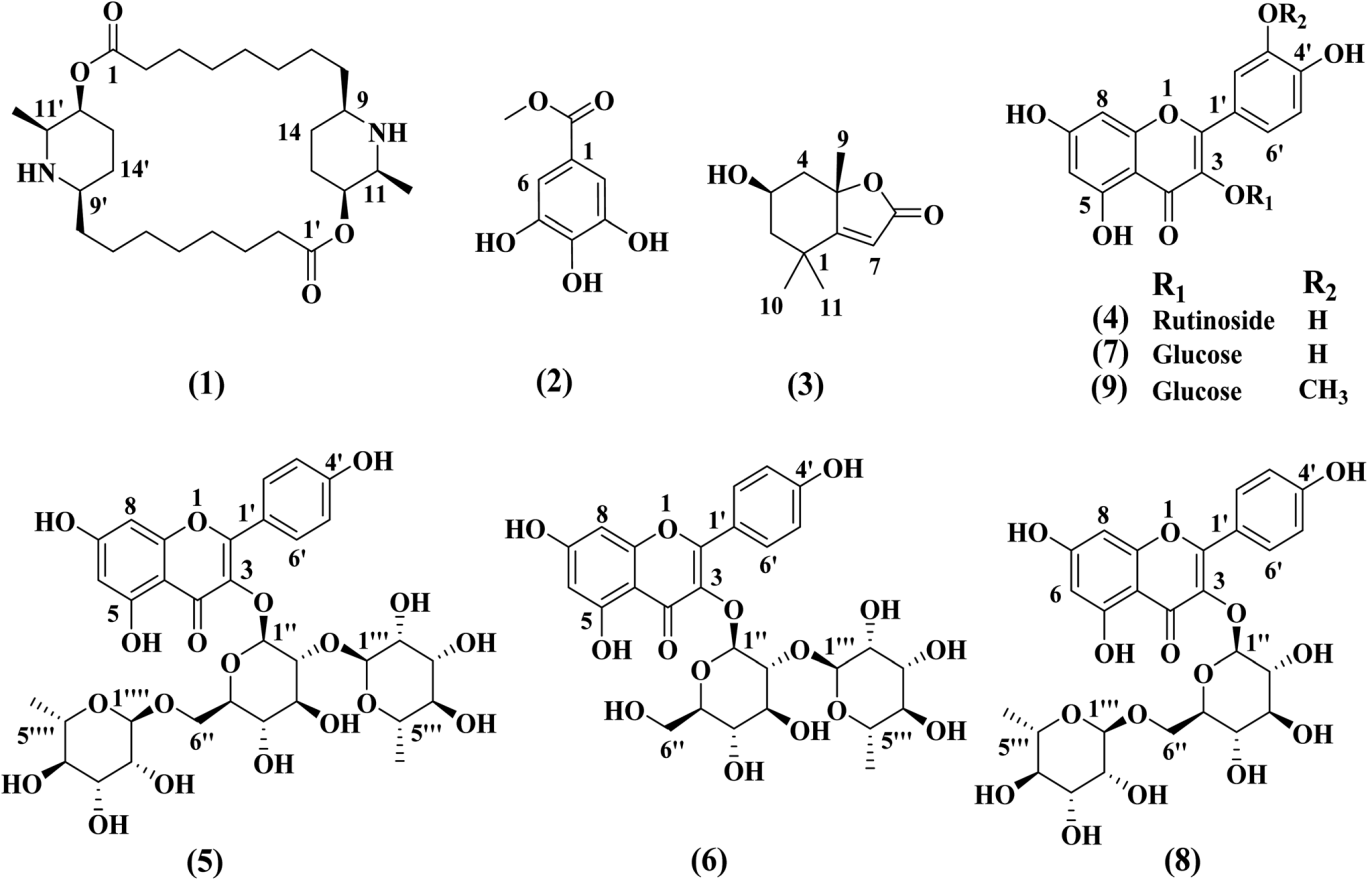
Chemical structures of the isolated compounds (1–9).

The chemical structures of compounds (1–9) was determined based on different spectroscopic data including 1D and 2D NMR, MS data, as well as comparison of the data with the previously reported in the literature. The structures of all the isolated compounds are declared in Fig. 1. The compounds were elucidated as carpaine (1),^19^ methyl gallate (2),^20^ loliolide (3),^21^ rutin (4),^22^ clitorin (5),^23^ kaempferol-3-*O*-neohesperidoside (6),^24^ isoquercetin (7),^25^ nicotiflorin (8)^26^ and isorhamnetin-3-*O*-β-d-glucopyranoside (9).^27^ The compounds 3, 4, 5–7 and 9 were isolated for the first time from the genus *Carica*.

### 2.2. MTT cytotoxicity assay in breast cancer cells line (MCF-7)

Breast cancer is a common malignancy among females linked with several risk factors as age, familial factors, reproductive factors, lifestyle and hormonal factors. Breast cancer usually starts from ductal hyperproliferation and then develops into either benign or metastatic tumors influenced by exposure to carcinogens.^28^ Extensive studies investigated the role of dietary and natural products in the reduction of development and progression of breast tumors. Natural products exhibit several anticancer activities by different mechanism such as direct inhibition of tumor cell proliferation, metastasis and angiogenesis of breast tumor cells.^29^

The cytotoxic effect of the isolated compounds from *C. papaya* on MCF-7 cells line was evaluated using MTT assay which measures metabolic activity as an indicator of cellular viability and proliferation. The results (Table 1) showed that methyl gallate (2) and clitorin (5) exhibited the most potent cytotoxic effects against MCF-7 cell lines with the IC_50_ value 1.11 ± 0.06 and 2.47 ± 0.14 μM, respectively. Moreover, kaempferol-3-*O*-neohesperidoside (6), nicotiflorin (8) and isorhamnetin-3-*O*-β-d-glucopyranoside (9) showed strong effect with IC_50_ values higher than the standard drug staurosporine (IC_50_ = 10.2 ± 0.58) (Table 1). The possible mechanism of their potential effect should be investigated for the development of targeted drug therapy for breast tumors.

**Table 1:** IC_50_ value of isolated compounds and staurosporine on MCF-7 cell lines

No.	Compound	IC_50_ (μM)
1	Carpaine	13.7 ± 0.78
2	Methyl gallate	1.11 ± 0.06
3	Loliolide	28.2 ± 1.61
4	Rutin	25.6 ± 1.46
5	Clitorin	2.47 ± 0.14
6	Kaempferol-3-*O*-neohesperidoside	3.58 ± 0.2
7	Isoquercetin	13.1 ± 0.75
8	Nicotiflorin	4.94 ± 0.28
9	Isorhamnetin-3-*O*-β-d-glucopyranoside	9.51 ± 0.54
	Staurosporine (positive control)	10.2 ± 0.58

### 2.3. EGFR^wt^ kinase activity

Herein, we studied the inhibitory activity of the isolated compounds on EGFR^wt^.^30,31^ The results (Table 2) revealed that all the tested compounds possess EGFR^wt^ potent inhibition activity at nM concentration with IC_50_ values ranging from 37.3 ± 1.9 to 100.20 ± 5.1 nM. Methyl gallate (2) and nicotiflorin (8) exhibited the highest inhibitory activities comparable with erlotinib (IC_50_ = 35.94 nM) with IC_50_ values of 37.3 ± 1.9 and 41.08 ± 2.1 nM, respectively. On the other hand, compounds 1, 3–7 and 9 had an interference effect with EGFR^wt^ but to lesser extent than methyl gallate (2) and nicotiflorin (8).

**Table 2:** *In vitro* enzymatic inhibitory activities of isolated compounds (1–9) against EGFR^wt^ and aromatase (CYP19A)

No.	Compound	IC_50_ (nM)	
		EGFR^wt^	Aromatase
1	Carpaine	47.59 ± 2.4	107.90 ± 6.32
2	Methyl gallate	37.30 ± 1.9	94.13 ± 5.51
3	Loliolide	68.82 ± 3.5	207.60 ± 12.2
4	Rutin	44.51 ± 2.3	147.60 ± 8.64
5	Clitorin	89.58 ± 4.6	77.41 ± 4.53
6	Kaempferol-3-*O*-neohesperidoside	64.46 ± 3.3	334.60 ± 19.6
7	Isoquercetin	83.40 ± 4.2	354.20 ± 20.7
8	Nicotiflorin	41.08 ± 2.1	92.84 ± 5.44
9	Isorhamnetin-3-*O*-β-d-glucopyranoside	100.20 ± 5.1	436.40 ± 25.6
	Erlotinib (positive control)	35.94 ± 1.8	
	Letrozole (positive control)		77.72 ± 4.55

### 2.4. Aromatase (CYP19A) enzyme activity

Breast cancer is mostly reliant on estrogen or progesterone, especially in postmenopausal females. Generally, there are two common treatment approaches for breast cancer, modulation of estrogen receptor by selective estrogen receptor modulators or inhibition of aromatase enzyme by aromatase inhibitors. Aromatase is the key enzyme that acts on androgen precursors for the synthesis of estrogen. Aromatase inhibitors are used to either block the production of estrogen or block the action of estrogen on its receptors and for treatment of estrogen dependant breast cancer.^32,33^ In the current study, the investigation of the effect of isolated compounds on aromatase (CYP19A) revealed that all the tested compounds potently inhibited the effect of aromatase enzyme with IC_50_ range from 77.41 ± 4.53 to 436.40 ± 25.6 nM (Table 2).

**Fig. 2:**
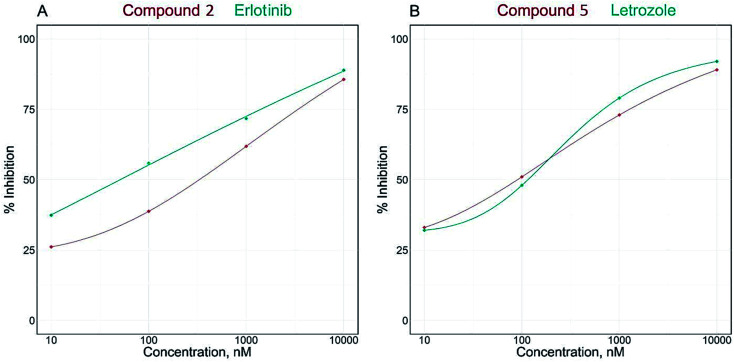
IC_50_ dose response curve of enzymatic inhibitory activities of isolated compounds 2 and 5 against EGFR^wt^ (A) and aromatase (CYP19A) (B).

The IC_50_ values of compounds clitorin (5) and nicotiflorin (8) were 77.41 ± 4.53 and 92.84 ± 5.44 nM, respectively. The results are demonstrated in Fig. 2 and Table 2. It is noteworthy that clitorin (**5**) was more effective than the standard drug letrozole (IC_50_ = 77.72 ± 4.55). In addition, the nicotiflorin (8) showed a dual potent effect on both EGFR and aromatase comparable to the standard drugs which could be scaffold to the development of safe effective therapy for breast cancer.

### 2.5. Molecular docking study

**Fig. 3:**
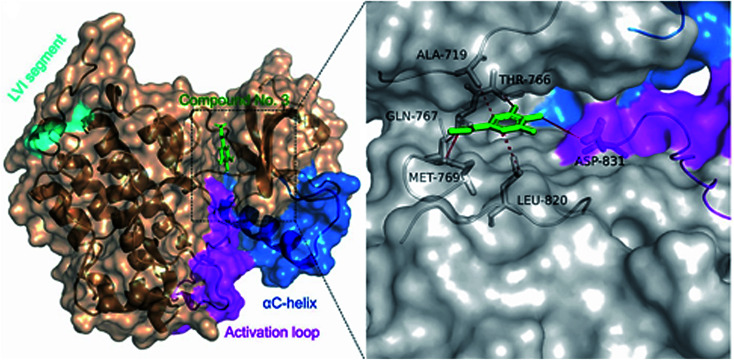
Ligand interactions of metyl gallate (2) with EGFR receptor.

In order to understand the binding mode of the most potent compounds with the tested enzymes, a molecular docking analysis was conducted. EGFR kinase domain includes essential regulatory elements including αC helix (amino acids 729–744) and activation loop (amino acids 831–852).^34^ These elements are important for allosteric regulation and conformational changes and controlling EGFR activation. Our molecular docking analysis showed that methyl gallate (2) bound with the EGFR (PDB ID: 1M17) through hydrogen bonding with MET-769, THR-766, GLN-767 amino acid residues. In addition, methyl gallate (2) bound with ASP-831 in the activation loop of EGFR kinase domain. The binding pose score was −4.5287 kcal mol^−1^ with a root mean square deviation (RMSD) value of 1.69 Å in comparison to standard inhibitor erlotinib (−6.7615 kcal mol^−1^, RMSD = 1.24). Other hydrophobic interactions are shown in (Fig. 3).

**Fig. 4:**
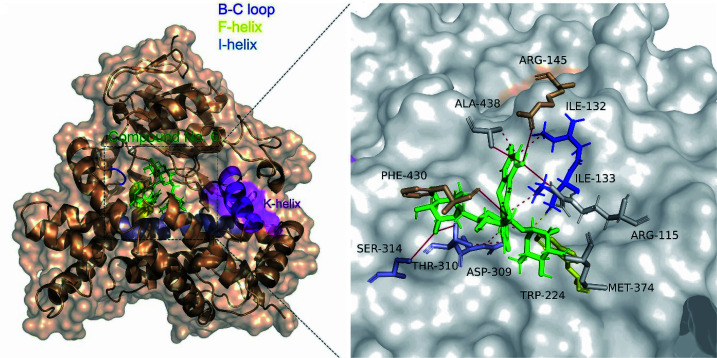
Ligand interactions of clitorin (5) with aromatase cytochrome P450 (CYP19A1) receptor.

The catalytic cleft of aromatase comprises amino acids Ile 305, Ala 306, Asp 309 and Thr 310 from the I-helix, Phe 221 and Trp 224 from the F-helix, Ile 133 and Phe 134 from the B–C loop, Val 370, Leu 372 and Val 373 from the K-helix-β3 loop, Met 374 from β3, Leu 477 and Ser 478 from the β8–β9 loop.^35^ Molecular docking analysis showed that clitorin (5) interacted with human aromatase cytochrome P450 (CYP19A1) (PDB ID: 3S79) with a posing score −14.2074 kcal mol^−1^ with RMSD value of 1.56 in comparison to standard inhibitors erlotinib (−11.2837 kcal mol^−1^, RMSD = 1.24) and letrozol (−7.2807 kcal mol^−1^, RMSD = 1.28). Clitorin (5) interacted through H-bonds with several amino acids in the catalytic cleft of aromatase. It formed H-bonds with ARG-145, ALA-438, PHE-430, ASP-309, SER-314 in the catalytic cleft I-helix, MET 374 and MET 311 (2 H-bonds) as hydrogen donors, while interacted as hydrogen acceptor with CYS 437 and MET 374 amino acid residues. It also interacted through hydrophobic interaction with ILE-133 from the BC-loop in the catalytic cleft (Fig. 4).

### 2.6. ADME pharmacokinetics and drug-likeness properties

The nine identified compounds were screened for their ADME pharmacokinetics and drug-likeness using the websites servers^36,37^ as previously described^38^ (Table 3). All tested compounds had good membrane permeability (log *p* values ≤5). The compounds (1–3) possessed good bioavailability scores with 0.55 value. Furthermore, compounds (4–9), showed promising drug-likeness scores. Detailed molecular properties, absorption, distribution, metabolism and excretion *in silico* assessment are shown in Table 3.

**Table 3:** Detailed drug likeness, molecular properties, absorption, distribution, metabolism and excretion *in silico* assessment

Molecule	1	2	3	4	5	6	7	8	9
MW	478.72	184.15	196.25	610.52	740.66	594.52	464.38	594.52	478.4
TPSA	76.66	86.99	46.53	269.43	308.12	249.2	210.51	249.2	199.51
MLOGP	3.75	0.18	1.49	−3.89	−4.77	−3.43	−2.59	−3.43	−2.37
No. atoms	34	13	14	43	52	42	33	42	34
nON	6	5	3	16	19	15	12	15	12
nOHNH	2	3	1	10	11	9	8	9	7
No. rotb	0	2	0	6	8	6	4	6	5
Fraction Csp3	0.93	0.12	0.73	0.44	0.55	0.44	0.29	0.44	0.32
Rotatable bonds	0	2	0	6	8	6	4	6	5
H-Bond acceptors	6	5	3	16	19	15	12	15	12
H-Bond donors	2	3	1	10	11	9	8	9	7
Molvolume	497.37	152.63	187.48	496.07	611.91	488.05	372.21	488.05	389.73
Lipinski violations	0	0	0	3	3	3	2	3	2
Ghose violations	2	0	0	4	4	4	1	4	0
Veber violations	0	0	0	1	1	1	1	1	1
Egan violations	0	0	0	1	1	1	1	1	1
Muegge violations	1	1	1	4	5	3	3	3	3
ESOL class	Poorly soluble	Very soluble	Very soluble	Soluble	Soluble	Soluble	Soluble	Soluble	Soluble
GI absorption	High	High	High	Low	Low	Low	Low	Low	Low
BBB permeant	No	No	Yes	No	No	No	No	No	No
Pgp substrate	Yes	No	No	Yes	No	Yes	No	Yes	Yes
CYP1A2 inhibitor	No	No	No	No	No	No	No	No	No
CYP2C19 inhibitor	No	No	No	No	No	No	No	No	No
CYP2C9 inhibitor	No	No	No	No	No	No	No	No	No
CYP2D6 inhibitor	No	No	No	No	No	No	No	No	No
CYP3A4 inhibitor	No	No	No	No	No	No	No	No	No
BBB score	2.48	2.68	3.68	1.21	1	1.24	1.61	1.24	1.57
Bioavailability score	0.55	0.55	0.55	0.17	0.17	0.17	0.17	0.17	0.17
Drug-likeness model score	−1.49	−0.65	−1.02	0.91	0.9	0.88	0.68	0.9	0.59

## 3. Experimental section

### 3.1. General experimental procedures

^1^H- and ^13^C-NMR spectra were acquired at 25 °C using a Varian Inova 400 MHz NMR spectrometer. High-resolution mass spectra were measured using a Thermo scientific LTQ/XL Orbitrap, with FTMS analyzer, a mass range of 100–2000 and a resolution of 30 000. For LC-ESI-MS, gradient separation was obtained using a Sun Fire C-18 analytical HPLC column (5 mm, 4.6 × 150 mm, Waters) with a mobile phase of 0–100% MeOH over 30 min at a flow rate of 1 mL min^−1^. HPLC separation was performed on Agilent 1260 Infinity semi-preparative HPLC system with an Agilent Eclipse XDB-C18 column (5 μm, 10 × 250 mm, Agilent technologies, USA) monitored using an Agilent photodiode array detector. Detection was carried out at 220, 254, 280, 350 and 400 nm. All chemical reagents were purchased from Sigma-Aldrich (USA) and used without further purification. Medium pressure liquid chromatography (MPLC) separations were carried out using Biotage system with normal silica and reversed-phase pre-packed columns. UV-Detection was carried out at 220 and 254 nm. TLC was performed on pre-coated TLC plates with silica gel 60 F_254_ (layer thickness 0.2 mm, Merck, Darmstadt, Germany).

### 3.2. Plant material

*C. papaya* leaves were collected in June 2019 from the Research Farm, Faculty of Agriculture, Minia University, Minia, Egypt. The plant was identified by Prof. Mahmoud A. H. Abdou, Department of Horticulture, Faculty of Agriculture, Minia University. A voucher sample (Mn-Ph-Cog-044) was kept in the Herbarium of the Pharmacognosy Department, Faculty of Pharmacy, Minia University, Minia, Egypt. The plant materials were dried in shade, finely powdered and the powder was used for further analysis.

### 3.3. Extraction and isolation

The air-dried powdered leaves of *C. papaya* (1.8 kg) were extracted with MeOH–H_2_O (8 : 2, v/v, 4 × 8 L each) in a closed glass container at room temperature for three times at one week interveals. The total hydromethanolic extract (THME) was concentrated under reduced pressure at 40 °C to afford a dark yellowish-green residue (160 g). THME was suspended in distilled water (500 mL) and successively partitioned between *n*-hexane (500 mL, 4×), methylene chloride (MC) (500 mL, 4×) and ethyl acetate (EtOAc) (500 mL, 4×); each fraction was concentrated under reduced pressure to give *n*-hexane (80 g), MC (16 g), EtOAc (25 g) and aqueous (30 g), respectively. A part of MC fraction (10 g) was chromatographed on Biotage flash chromatography system using prepacked silica column using *n*-hexane–EtOAc gradients (10 : 0, 9 : 1, 8.5 : 1.5, 8 : 2, 7.5 : 2.5 and 7 : 3, v/v) to afford compound 1 (20 mg), compound 2 (40 mg) and compound 3 (50 mg).

Furthermore, a part of EtOAc fraction (10 g) was subjected to MPLC Biotage system using prepacked RP-18 column chromatography with a mobile phase H_2_O–MeOH gradient (9.5 : 0.5, 9 : 1, 8.5 : 1.5, 8 : 2, 7.5 : 2.5 and 7 : 3), v/v to afford four major fractions; fraction A (1.8 g), fraction B (2.6 g), fraction C (1.5 g) and fraction D (1.4 g). Fraction B was subjected to semi-preparative HPLC to afford compound 4 (8 mg), compound 5 (6 mg), compound 6 (3 mg), compound 7 (4 mg), compound 8 (5 mg) and compound 9 (7 mg).

Compound 1 (carpaine) was separated as a white crystalline. Its positive HR-ESI-MS: *m*/*z* 479.3828 [M + H]^+^ (calcd for C_28_H_51_N_2_O_4_: 479.3849). The ^1^H-NMR (400 MHz, CD_3_OD) *δ*_H_: 4.98 (2H, br s, H-12, H-12′), 3.48 (2H, q, *J* = 6.4 Hz, H-11, H-11′), 3.18 (2H, m, H-9, H-9′), 2.36–2.52 (4H, m, H-2, H-2′), 1.81–2.01 (4H, m, H-13, H-13′), 1.68–1.78 (4H, m, H-14, H-14′), 1.68–1.78 (4H, m, H-3, H-3′), 1.68–1.78 (4H, m, H-7, H-7′), 1.34–1.43 (4H, m, H-4, H-4′), 1.34–1.43 (4H, m, H-5, H-5′), 1.34–1.43 (4H, m, H-6, H-6′), 1.34–1.43 (4H, m, H-8, H-8′), and 1.28 (6H, d, *J* = 6.7 Hz, CH_3_-11, 11′). ^13^C-NMR (100 MHz, CD_3_OD): *δ*_C_: 174.2 (C-1, C-1′), 69.2 (C-12, C-12′), 58.3 (C-9, C-9′), 55.6 (C-11, C-11′), 34.9 (C-2, C-2′), 34.2 (C-8, C-8′), 29.9 (C-6, C-6′), 29.3 (C-5, C-5′), 29.0 (C-4, C-4′), 27.9 (C-13, C-13′), 26.3 (C-7, C-7′), 25.5 (C-14, C-14′), 24.0 (C-3, C-3′) and 15.7 (CH_3_ attached to C-11, C-11′). The spectral data of compound 1 are shown in Fig. S1–S6.†

Compound 2 (methyl gallate) was separated as a whitish-grey powder. The ^1^H-NMR (400 MHz, CD_3_OD) *δ*_H_: 3.81 (3H, s, OCH_3_-1) and 7.04 (2H, s, H-2, H-6). The ^1^H-NMR spectrum is illustrated in Fig. S7.†

Compound 3 (loliolide) was separated as a white amorphous powder. The positive HR-ESI-MS: *m*/*z* 197.1179 [M + H]^+^ (calcd for C_11_H_16_O_3_: 197.1177). The ^1^H-NMR (400 MHz, CD_3_OD) *δ*_H_: 5.75 (1H, s, H-7), 4.22 (1H, m, H-3), 2.48 (1H, dt, *J* = 13.5, 2.4 Hz, H-4a), 1.99 (1H, dt, *J* = 14.4, 3.3 Hz, H-2a), 1.76 (3H, s, CH_3_), 1.74 (1H, dd, *J* = 13.6, 3.8 Hz, H-4b), 1.55 (1H, dd, *J* = 14.4, 3.4 Hz, H-2b), 1.47 (3H, s, CH_3_) and 1.28 (3H, s, CH_3_). ^13^C-NMR (100 MHz, CD_3_OD) *δ*_C_: 185.7 (C-6), 174.4 (C-8), 113.3 (C-7), 88.9 (C-5), 67.2 (C-3), 48.0 (C-2), 46.5 (C-4), 37.2 (C-1), 31.0 (C-9, CH_3_), 27.4 (C-11, CH_3_) and 27.0 (C-10, CH_3_). The spectral data of compound 3 are demonstrated in Fig. S8–S13.†

Compound 4 [Rutin (syn.: quercetin-3-*O*-rutinoside or sophorin or rutoside)] was separated as a yellow powder. The positive HR-ESI-MS: *m*/*z* 611.1607 [M + H]^+^ (calcd for C_27_H_30_O_16_: 611.1612). The full spectral data are shown in Fig. S14–S18.† The ^1^H-NMR (400 MHz, DMSO-*d*_6_) spectral data of the aglycone displayed signals at *δ*_H_: 12.59 (1H, br s, OH–C5), 7.55 (1H, br s, H-2′), 7.54 (1H, d, *J* = 8.2 Hz, H-6′), 6.84 (1H, d, *J* = 8.2 Hz, H-5′), 6.38 (1H, d, *J* = 1.9 Hz, H-8), 6.19 (1H, d, *J* = 1.9 Hz, H-6); 3-glucosyl unit; 5.34 (d, *J* = 7.3 Hz, H-1′′) and rhamnosyl unit attached to C-6′′ of glucosyl unit; 4.38 (1H, br s, H-1′′′) and 0.99 (3H, d, *J* = 6.3 Hz, H-6′′′). ^13^C-NMR experiment (100 MHz, DMSO-*d*_6_), the aglycone showed signals at *δ*_C_: 177.3 (C-4), 164.1 (C-7), 161.2 (C-5), 156.6 (C-2), 156.4 (C-9), 148.4 (C-4′), 144.7 (C-3′), 133.3 (C-3), 121.7 (C-5′), 121.2 (C-1′), 116.5 (C-6′), 115.2 (C-2′), 103.9 (C-10), 98.7 (C-6), 93.6 (C-8), carbons of glucosyl unit; *δ*_C_ 101.2 (C-1′′), 76.3 (C-3′′), 75.9 (C-5′′), 74.1 (C-2′′), 70.1 (C-4′′) and 67.6 (C-6′′) and carbons of rahmnosyl unit attached to C-6′′ of glucosyl unit *δ*_C_: 100.7 (C-1′′′), 71.8 (C-4′′′), 70.6 (C-3′′′), 70.4 (C-2′′′), 68.2 (C-5′′′) and 17.7 (C-6′′′).

Compound 5 [clitorin (syn.: kaempferol 3-*O*-(2′′,6′′-di-*α-O*-rhamnopyranosyl)-β-glucopyranoside)] was separated as a yellowish powder. The positive HR-ESI-MS: *m*/*z* 741.2243 [M + H]^+^ (calcd for C_33_H_40_O_19_: 741.2242). 1D and 2D spectral data are illustrated in Fig. S19–S23.† The ^1^H-NMR spectral data (400 MHz, DMSO-*d*_6_) the aglycone displayed signals at *δ*_H_: 12.64 (1H, br s, OH–C5), 7.95 (2H, d, *J* = 8.7, H-2′, H-6′), 6.87 (2H, d, *J* = 8.8, H-3′, H-5′), 6.40 (1H, d, *J* = 2.0 Hz, H-8), 6.19 (1H, d, *J* = 2.0 Hz, H-6); 3-glucosyl unit; 5.48 (d, *J* = 7.0 Hz, H-1′′), 3.66 (1H, m, H-6′′a), 3.22 (1H, m, H-6′′b), rhamnosyl unit attached to C-2′′ of glucosyl unit; 5.05 (1H, br s, H-1′′′), 0.96 (3H, d, *J* = 6.4 Hz, H-6′′′) and rhamnosyl unit attached to C-6′′ of glucosyl unit; 4.32 (1H, br s, H-1′′′) and 0.81 (3H, d, *J* = 6.4 Hz, H-6′′′). ^13^C-NMR experiment (100 MHz, DMSO-*d*_6_), the aglycone showed signals at *δ*_C_: 177.2 (C-4), 164.1 (C-7), 161.2 (C-5), 159.8 (C-4′), 156.9^a^ (C-9), 156.4^a^ (C-2), 132.6 (C-3), 130.7 (C-2′), 130.7 (C-6′), 98.7 (C-6), 93.7 (C-8), 104.0 (C-10), 121.0 (C-1′), 115.1 (C-3′), 115.1 (C-5′), carbons of 3-glucosyl unit; *δ*_c_ 98.7 (C-1′′), 77.3^b^ (C-3′′), 77.1^b^ (C-5′′), 75.6 (C-2′′), 70.5^c^ (C-4′′) and 68.26^d^ (C-6′′), carbons of rhamnosyl unit attached to C-2′′ of glucosyl unit; *δ*_c_ 100.6^e^ (C-1′′′), 71.81^f^ (C-4′′′), 70.5^c^ (C-3′′′), 70.3^c^ (C-2′′′), 68.30^d^ (C-5′′′) and 17.3^g^ (C-6′′′), carbons of rhamnosyl unit attached to C-6′′ of glucosyl unit; *δ*_c_ 100.8^e^ (C-1′′′), 71.78^f^ (C-4′′′), 70.6^c^ (C-3′′′'), 70.3^c^ (C-2′′′), 68.3 (C-5′′′) and 17.7^g^ (C-6′′′), (^a^, ^b^, ^c^, ^d^, ^e^, ^f^ and ^g^ signals may be interchanged).

Compound 6 [kaempferol-3-*O*-neohesperidoside (syn.: kaempferol-3-*O*-glucorhamnoside or kaempferol 3-*O*-(2′′-*O-α*-l-rhamnopyranosyl)-β-d-glucopyranoside)] was separated as a yellow amorphous powder. The positive HR-ESI-MS: *m*/*z* 595.1659 [M + H]^+^ (calcd for C_27_H_31_O_15_: 595.1657). The ^1^H-NMR and positive HR-ESI-MS spectral data are shown in Fig. S24–S25.† The ^1^H-NMR spectral data (400 MHz, DMSO-*d*_6_) of the aglycone displayed signals at *δ*_H_: 12.64 (1H, br s, OH–C-5), 8.04 (2H, d, *J* = 8.8 Hz, H-2′, H-6′), 6.88 (2H, d, *J* = 8.8 Hz, H-3′, H-5′), 6.43 (1H, d, *J* = 2.0 Hz, H-8), 6.20 (1H, d, *J* = 2.0 Hz, H-6); 3-glucosyl unit; 5.65 (d, *J* = 7.2 Hz, H-1′′), 3.67 (1H, m, H-6′′a), 3.48 (1H, m, H-6′′b) and rhamnosyl unit attached to C-2′′ of glucosyl unit; 4.13 (1H, br s, H-1′′′) and 0.75 (3H, d, *J* = 6.0, H-6′′′).

Compound 7 [isoquercetin (syn.: quercetin-3-*O*-β-d-glucopyranoside or isoquercitrin or isotrifoliin)] was separated as a yellow powder. The positive HR-ESI-MS: *m*/*z* 465.1028 [M + H]^+^ (calcd for C_21_H_21_O_12_: 465.1027). The ^1^H-NMR and positive HR-ESI-MS spectral data are shown in Fig. S26–S27.† The ^1^H-NMR spectral data (400 MHz, DMSO-*d*_6_) of the aglycone displayed signals at *δ*_H_: 12.63 (1H, br s, OH–C5), 7.58 (2H, m, H-2′, H-6′), 6.84 (1H, d, *J* = 8.8 Hz, H-5′), 6.40 (1H, d, *J* = 2.0 Hz, H-8), 6.20 (1H, d, *J* = 2.0 Hz, H-6); 3-glucosyl unit; 5.46 (d, *J* = 7.6 Hz, H-1′′) 3.57 (1H, m, H-6′′a), 3.43 (1H, m, H-6′′b), 3.26 (2H, m, H-2′′, H-3′′) and 3.09 (2H, m, H-4′′, H-5′′).

Compound 8 [nicotiflorin (syn.: kaempferol-3-*O*-rutinoside or nicotifloroside or nictoflorin)] was separated as a yellow amorphous powder. The positive HR-ESI-MS: *m*/*z* 595.1661 [M + H]^+^ (calcd for C_27_H_30_O_16_: 595.1663). The ^1^H-NMR and positive HR-ESI-MS spectral data are shown in Fig. S28–S29.† The ^1^H-NMR (400 MHz, DMSO-*d*_6_) spectral data of the aglycone displayed signals at *δ*_H_: 12.55 (1H, br s, OH–C5), 7.98 (2H, d, *J* = 8.8 Hz, H-2′, H-6′), 6.87 (2H, d, *J* = 8.8 Hz, H-3′, H-5′), 6.41 (1H, d, *J* = 1.6 Hz, H-8), 6.20 (1H, d, *J* = 1.6 Hz, H-6); 3-glucosyl unit; 5.31 (d, *J* = 7.6 Hz, H-1′′), 3.66–3.33 (2H, m, H-6) and rhamnosyl unit attached to C-6′′ of glucosyl unit; 4.37 (1H, br s, H-1′′′) and 0.96 (3H, d, *J* = 6.0 Hz, H-6′′′).

Compound 9 (isorhamnetin 3-*O*-β-d-glucopyranoside) was separated as a yellow amorphous powder. The positive HR-ESI-MS: *m*/*z* 479.1190 [M + H]^+^ (calcd for C_22_H_22_O_12_: 479.1189). 1D and 2D spectral data are illustrated in Fig. S30–S34.† The ^1^H-NMR spectral data (400 MHz, DMSO-*d*_6_) of the aglycone displayed signals at *δ*_H_: 12.61 (1H, br s, OH–C5), 7.94 (1H, d, *J* = 2.0 Hz, H-2′), 7.49 (1H, dd, *J* = 8.4, 2.0 Hz, H-6′), 6.92 (1H, d, *J* = 8.4 Hz, H-5′), 6.45 (1H, d, *J* = 2.0 Hz, H-8), 6.21 (1H, d, *J* = 2.0 Hz, H-6), 3.84 (3H, s, OCH_3_-3′); 3-glucosyl unit; 5.57 (d, *J* = 7.2 Hz, H-1′′), 3.57 (1H, m, H-6′′a), 3.39 (1H, m, H-6′′b), 3.23 (2H, m, H-2′′, H-3′′) and 3.11 (2H, m, H-4′′, H-5′′). ^13^C-NMR experiment (100 MHz, DMSO-*d*_6_), the aglycone showed signals at *δ*_C_: 177.4 (C-4), 164.2 (C-7), 161.2 (C-5), 156.4 (C-9), 156.3 (C-2), 149.4 (C-4′), 146.9 (C-5′), 133.0 (C-3), 122.0 (C-2′), 121.1 (C-1′), 115.2 (C-3′), 113.5 (C-6′), 104.0 (C-10), 98.7 (C-6), 93.7 (C-8), 55.7 (OCH_3_), carbons of 3-glucosyl unit; *δ*_c_ 100.8 (C-1′′), 77.5 (C-5′′), 76.4 (C-3′′), 74.3 (C-2′′), 69.8 (C-4′′) and 60.6 (C-6′′).

### 3.4. MTT cytotoxicity assay in breast cancer cells line (MCF-7)

The cell viability assay was performed to evaluate the effect of the isolated compounds (1–9) from *C. papaya* leaves on breast cancer cell line (MCF-7) using the *in vitro* MTT assay protocol as previously described by Hamed *et al.*, 2021.^39^ Briefly, breast cancer cell line (MCF-7) were obtained from American Type Culture Collection and cultured in DMEM medium (Invitrogen/Life Technologies) supplemented with 10% FBS (Hyclone, USA). Insulin (10 μg mL^−1^, Sigma-Aldrich, USA) and 1% penicillin–streptomycin. the cells were seeded in 96-well plates (VWR, Switzerland) at cell density 1.2–1.8 × 10^4^ cells per well. Then cells were treated with the isolated compounds (1–9) at serial concentrations in triplicates for 24 h. DMSO (0.1%, w/v) was used as a blank control and staurosporine was used as a positive control. After treatments, the medium was removed from all wells of the plate and 20 μL of the MTT reagent (Sigma-Aldrich, USA) (0.5 mg mL^−1^) in PBS was added and incubated for 3.5 h at 37 °C. The solution was removed and 50 μL of DMSO was added, incubated for 5 min and the absorbance was measured at 570 nm.

### 3.5. EGFR^wt^ kinase inhibition activity

Evaluation of the inhibitory activity of the isolated compounds (1–9) against EGFR^wt^ kinase was carried out using EGFR^wt^ Kinase Assay Kit (BPS biosciences) according to manufacturer's instructions.^40,41^ In brief, the master mixture was constructed from EGFR^wt^ enzyme, their substrates, ATP and kinase assay enzymatic buffer were incubated with the tested compounds for 40 min at 30 °C to achieve the enzymatic reaction. Then, the reaction was stopped by the addition of a detecting reagent (Kinase-Glo Max reagent), followed by incubation at room temperature for another 15 min. Finally, luminescence was measured using the microplate Robonik P2000 ELISA Reader. All samples and controls were tested in duplicate and the results are presented as percentage enzyme inhibition and compared to erlotinib selected as reference drugs due to their potent inhibitory activity of EGFR^wt^.

### 3.6. Aromatase (CYP19A) inhibition activity

The *in vitro* aromatase inhibitory activity of the compounds (1–9) was evaluated using (Bio Vision, Aromatase (CYP19A) Inhibitor Screening Kit (Fluorometric)) in comparison to letrozole as the reference drug. This method was carried out according to Acar Çevik *et al.*, 2020 procedure.^42^ The compounds were dissolved in DMSO in concentrations ranging from 10 to 10^4^ nM. The recombinant human aromatase stock was prepared according to the protocol and the samples were added and the plate was incubated for 10 min at 37 °C to allow tested compounds to interact with the aromatase. After incubation, 30 μL of the aromatase substrate/NADP^+^ mixture was added to each well and the fluorescence at *E*_x_/*E*_m_ = 488/527 nm was measured immediately (within 1 min).

### 3.7. Molecular docking simulation

Molecular docking was performed as briefly described to understand the binding affinity of the potent compounds in comparison with the reference drugs at the molecular level.^43^ The crystal structure of both the epidermal growth factor receptor tyrosine kinase (EGFR^wt^) (PDB ID: 1M17) and Human placental aromatase cytochrome P450 (CYP19A1) for the breast cancer receptor (PDB ID: 3S79) were obtained from the Protein data bank. The molecular docking simulation was conducted using the “Molecular Operating Environment (MOE 2014.9) and preparation and optimization of both ligands and receptors were carried out according to induced fit MOE protocol.^44^ The parameters of scoring were Triangle Matcher, scoring was set at London dG and rescoring at GBVI/WSA dG. The docking score, root mean square deviation (RMSD) and ligand–receptor complexes were tested for interaction analysis. The 3D images were made using the MOE visualizing tool.

### 3.8. Drug like properties and ADME prediction of isolated compounds

The drug likeliness, molecular properties prediction ADME and pharmacokinetic parameters of the isolated compounds were calculated using a set of software including “MolSoft,” “Molinspiration”, “PreADME” and “SwissADME” websites servers.^36,37^

### 3.9. Statistical analysis

Results are expressed as mean ± standard deviation (SD) based on triplicate experiments. The IC_50_ values of the tested compounds were determined using curve fitting in the R programming language and associated packages including Magrittr,^45^ drc^46^ and ggplot2.^47^ Graphs and figures were generated using R console and PyMOL (PyMOL Molecular Graphics System, Schrödinger).

## 4. Conclusion

Due to the reported various secondary metabolites of *C. papaya* Linn. leaves, in addition to their promising cytotoxic activity against breast cancer cell line supported by protein kinase inhibition activities and molecular docking study. They could be considered as potential candidates against breast cancer. Therefore, further investigations could have a supportive role in the pharmaceutical field towards the development of new breast anti-cancer drugs.

## Conflicts of interest

The authors declare no conflict of interest.

## Supplementary Material

RA-012-D1RA07000B-s001
