# Supplementary material for: Chemical constituents from Carica papaya Linn. leaves as potential cytotoxic, EGFRwt and aromatase (CYP19A) inhibitors; a study supported by molecular docking
Source: RSC Adv. 2022 Mar 23;12(15):9154–62. doi: 10.1039/d1ra07000b (PMC8985094; doi:10.1039/d1ra07000b)
Supplement: RA-012-D1RA07000B-s001 [file RA-012-D1RA07000B-s001.pdf]

Electronic Supplementary Material (ESI) for RSC Advances.
This journal is © The Royal Society of Chemistry 2022

Supplementary Information for

Chemical constituents from *Carica papaya* Linn. leaves as potential cytotoxic, EGFR^{wt} and Aromatase (CYP19A) inhibitors; a study supported by molecular docking

Ashraf N. E. Hamed,[a] Mohamed E. Abouelela,[b] Ahmed E. El Zowalaty,[c,d,e] Mohamed M. Badr,[f] Mohamed S. A. Abdelkader,[g,*]

[a]Department of Pharmacognosy, Faculty of Pharmacy, Minia University, Minia 61519, Egypt.

[b]Department of Pharmacognosy, Faculty of Pharmacy, Al-Azhar University, Assiut-Branch, Assiut, 71524, Egypt.

[c]Sahlgrenska Center for Cancer Research, Department of Surgery, Institute of Clinical Sciences, University of Gothenburg, 40530 Gothenburg, Sweden.

[d]Wallenberg Centre for Molecular and Translational Medicine, University of Gothenburg, 40530 Gothenburg, Sweden.

[e]Department of Pharmacology and Toxicology, Faculty of Pharmacy, Zagazig University, 44519, Egypt.

[f]Department of Biochemistry, Faculty of Pharmacy, Menoufia University, Menoufia, 32511, Egypt.

[g]Department of Pharmacognosy, Faculty of Pharmacy, Sohag University, Sohag, 82524, Egypt.

***Corresponding Author:**

Mohamed S.A. Abdelkader: m.salaheldin@pharm.sohag.edu.eg

Contents

Figure	Page
**Fig. S1** ^{1}H-NMR spectrum of compound **1** (400 MHz, CD_3OD).	4
**Fig. S2** ^{13}C-NMR spectrum of compound **1** (100 MHz, CD_3OD).	4
**Fig. S3** HSQC spectrum of compound **1**.	5
**Fig. S4.** ^{1}H-^{1}H COSY spectrum of compound **1**.	5
**Fig. S5** HMBC spectrum of compound **1**.	6
**Fig. S6** Positive HR-ESI-MS spectrum of compound **1**.	6
**Fig. S7** ^{1}H-NMR spectrum of compound **2** (400 MHz, CD_3OD).	7
**Fig. S8** ^{1}H-NMR spectrum of compound **3** (400 MHz, CD_3OD).	7
**Fig. S9** ^{13}C-NMR spectrum of compound **3** (100 MHz, CD_3OD).	8
**Fig. S10** HSQC spectrum of compound **3**.	8
**Fig. S11** HMBC spectrum of compound **3**.	9
**Fig. S12** ^{1}H-^{1}H COSY spectrum of compound **3**	9
**Fig. S13** Positive HR-ESI-MS spectrum of compound **3**.	10
**Fig. S14** ^{1}H-NMR spectrum of compound **4** (400 MHz, DMSO-d_6).	10
**Fig. S15** ^{13}C-NMR spectrum of compound **4** (100 MHz, DMSO-d_6).	11
**Fig. S16** HSQC spectrum of compound **4**.	11
**Fig. S17** HMBC spectrum of compound **4**.	12
**Fig. S18** Positive HR-ESI-MS spectrum of compound **4**.	12
**Fig. S19** ^{1}H-NMR spectrum of compound **5** (400 MHz, DMSO-d_6).	13
**Fig. S20** ^{13}C-NMR spectrum of compound **5** (100 MHz, DMSO-d_6).	13
**Fig. S21** HSQC spectrum of compound **5**.	14
**Fig. S22** HMBC spectrum of compound **5**.	14
**Fig. S23** Positive HR-ESI-MS spectrum of compound **5**.	15
**Fig. S24** ^{1}H-NMR spectrum of compound **6** (400 MHz, DMSO-d_6).	15
**Fig. S25** Positive HR-ESI-MS spectrum of compound **6**.	16
**Fig. S26** ^{1}H-NMR spectrum of compound **7** (400 MHz, DMSO-d_6).	16
**Fig. S27** Positive HR-ESI-MS spectrum of compound **7**.	17
**Fig. S28** ^{1}H-NMR spectrum of compound **8** (400 MHz, DMSO-d_6).	17

**Fig. S29** Positive HR-ESI-MS spectrum of compound **8**.	18
**Fig. S30** ^{1}H-NMR spectrum of compound **9** (400 MHz, DMSO-d_6).	18
**Fig. S31** ^{13}C-NMR spectrum of compound **9** (100 MHz, DMSO-d_6).	19
**Fig. S32** HSQC spectrum of compound **9**.	19
**Fig. S33** HMBC spectrum of compound **9**.	20
**Fig. S34** Positive HR-ESI-MS spectrum of compound **9**.	20

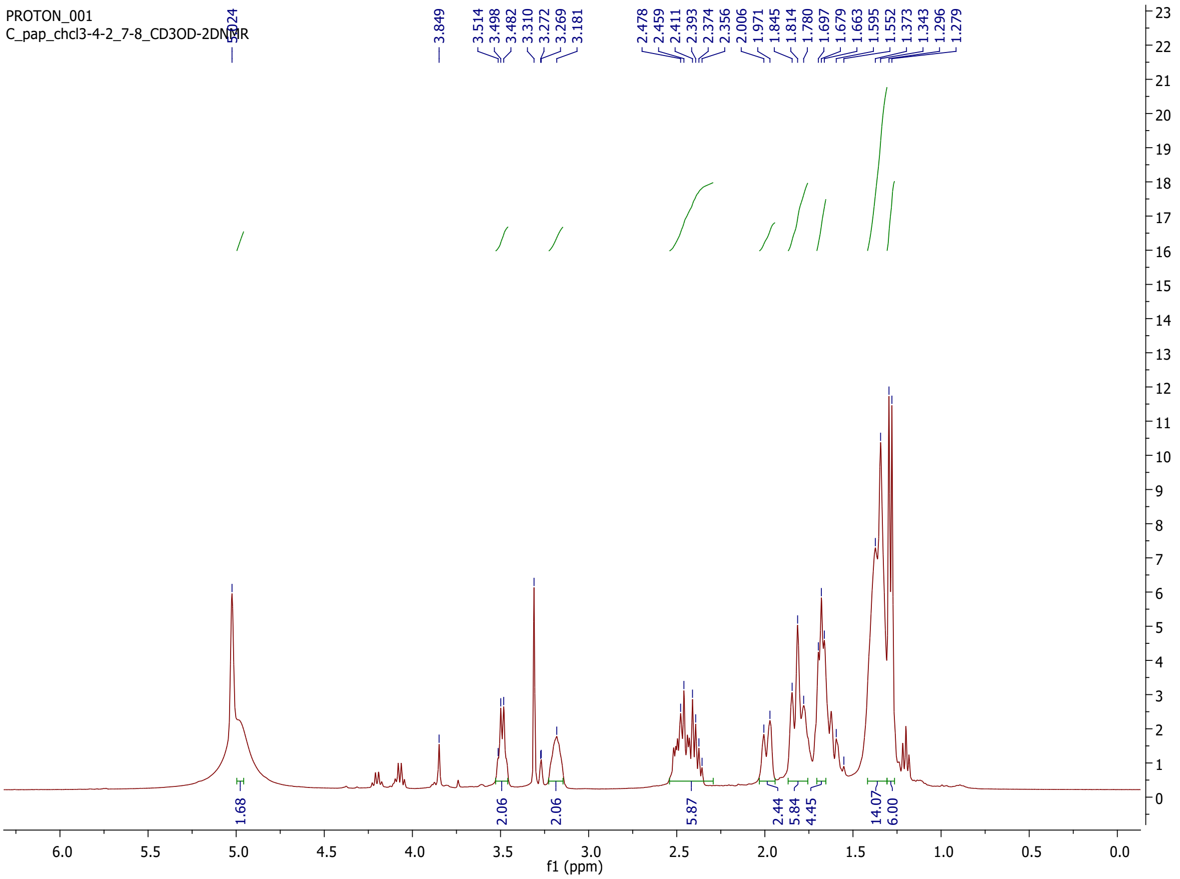

Fig. S1 ^{1}H-NMR spectrum of compound **1** (400 MHz, CD_3OD).

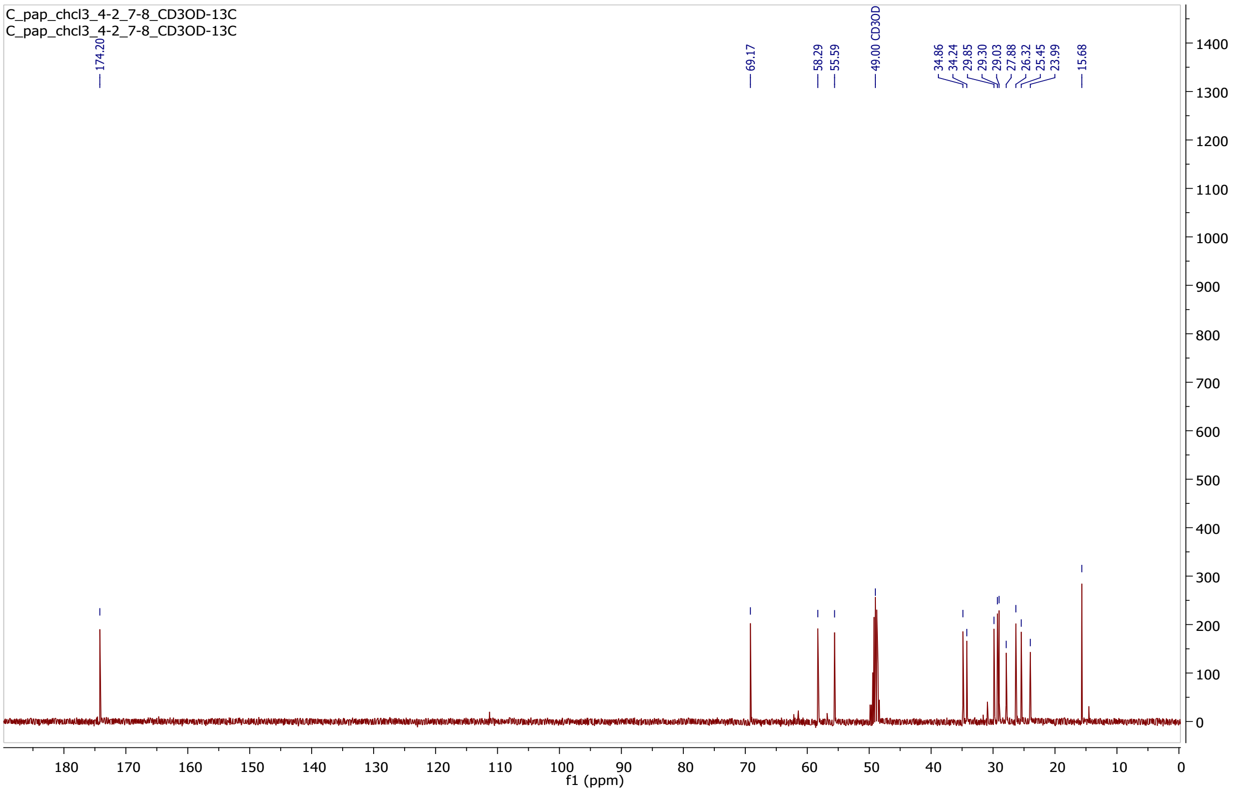

Fig. S2 ^{13}C-NMR spectrum of compound **1** (100 MHz, CD_3OD).

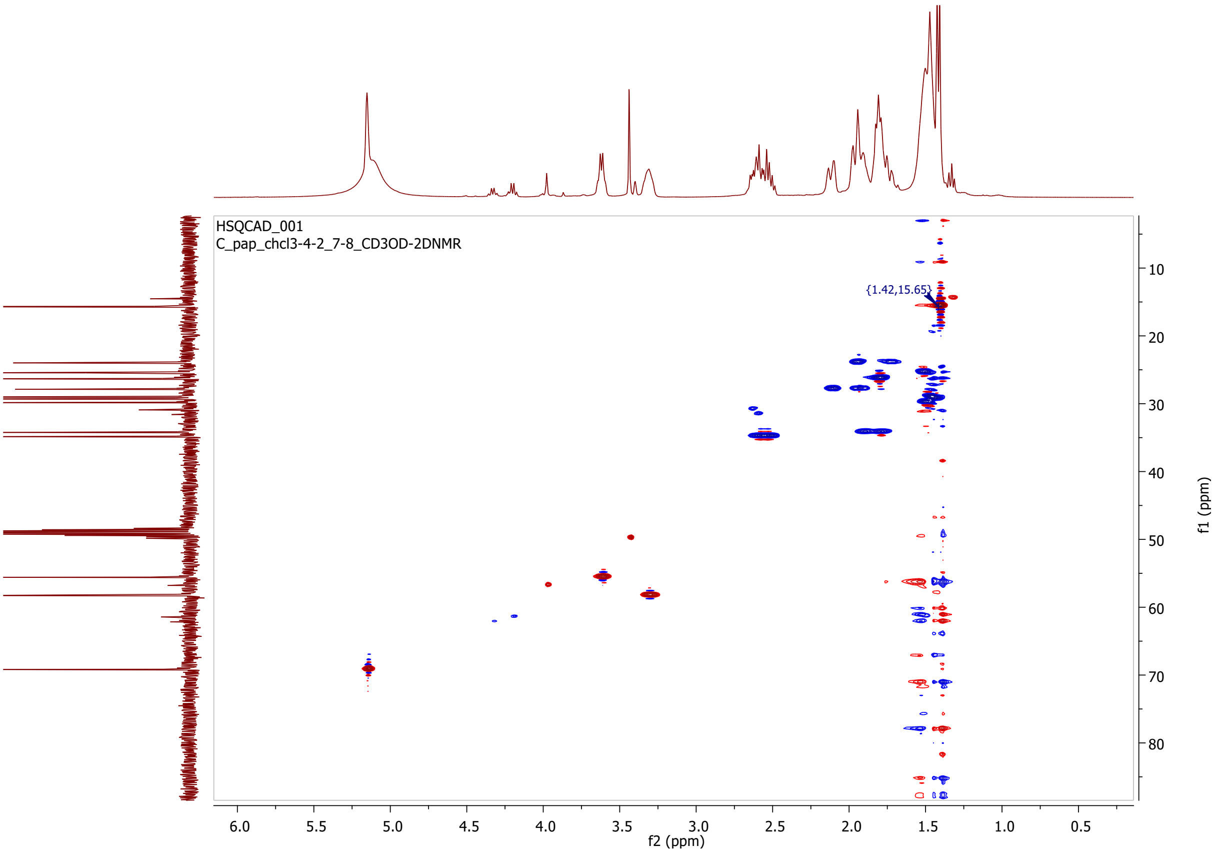

Fig. S3 HSQC spectrum of compound **1**.

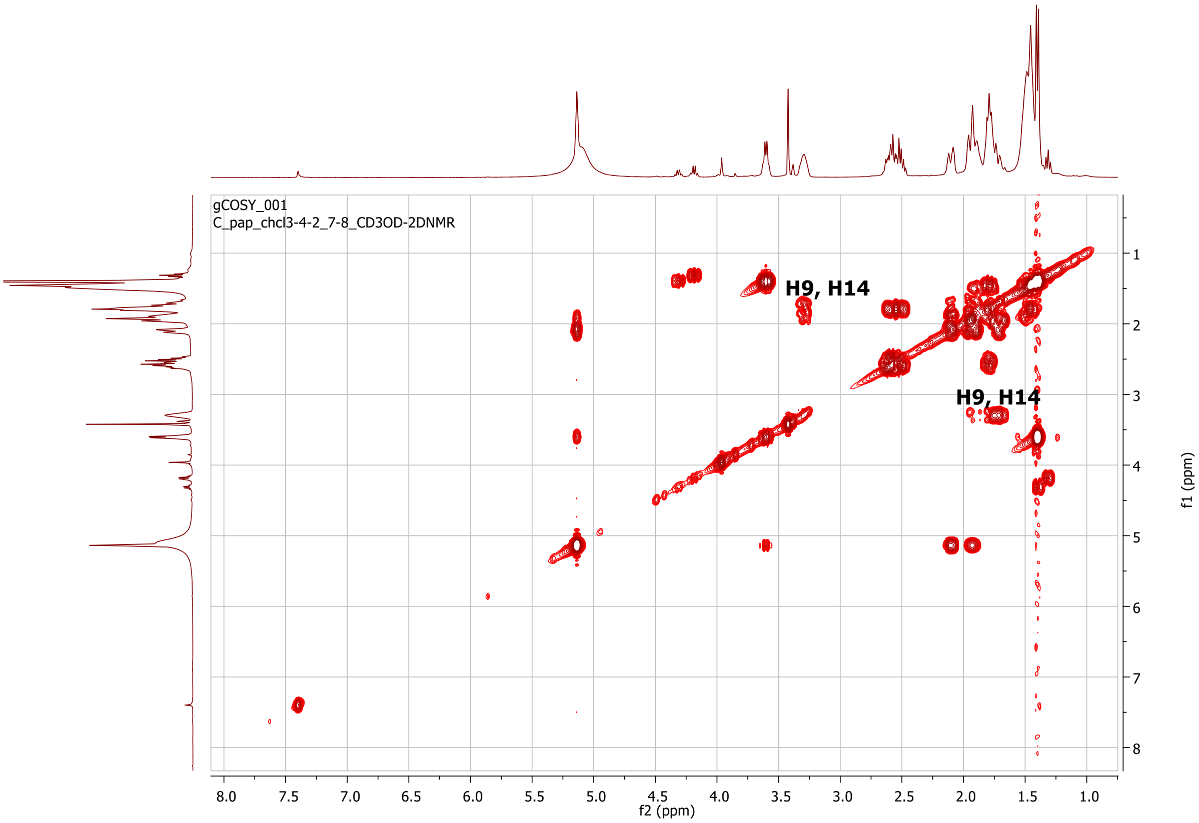

Fig. S4. ^{1}H-^{1}H COSY spectrum of compound **1**.

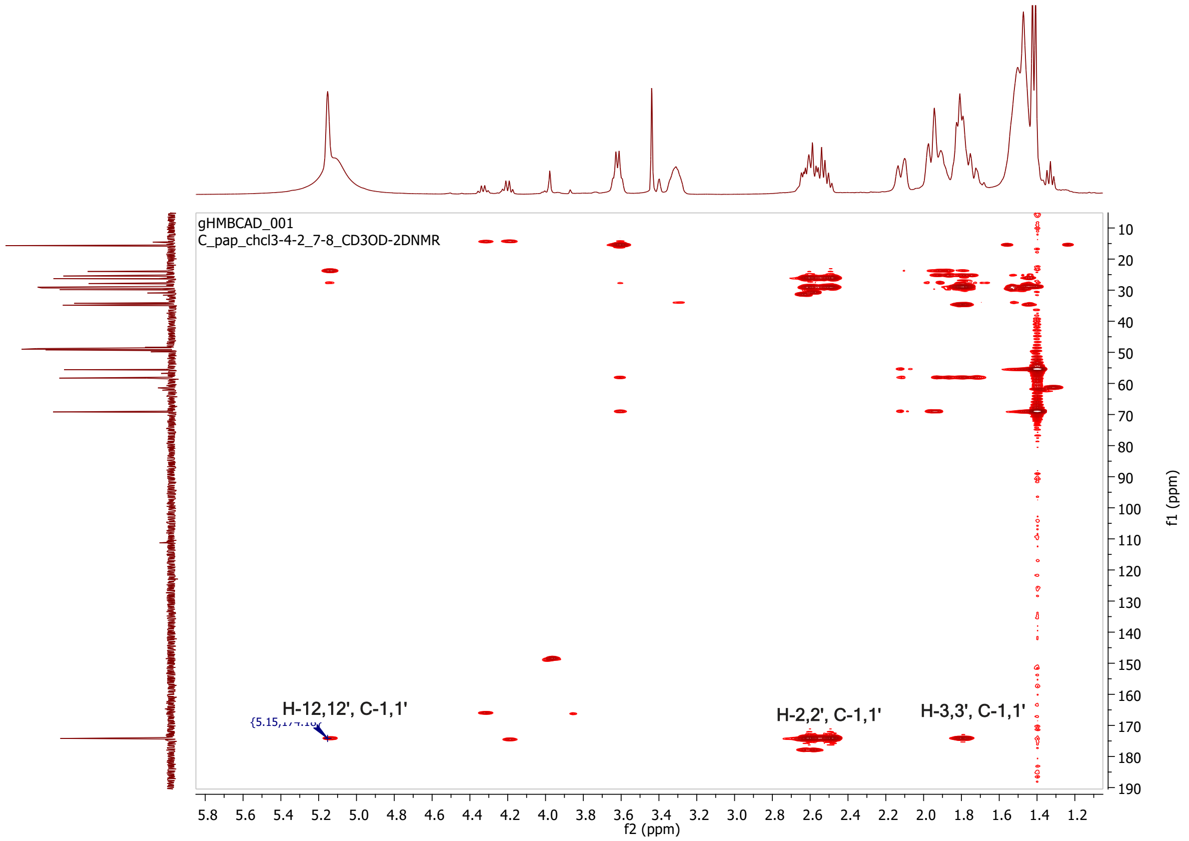

Fig. S5 HMBC spectrum of compound **1**.

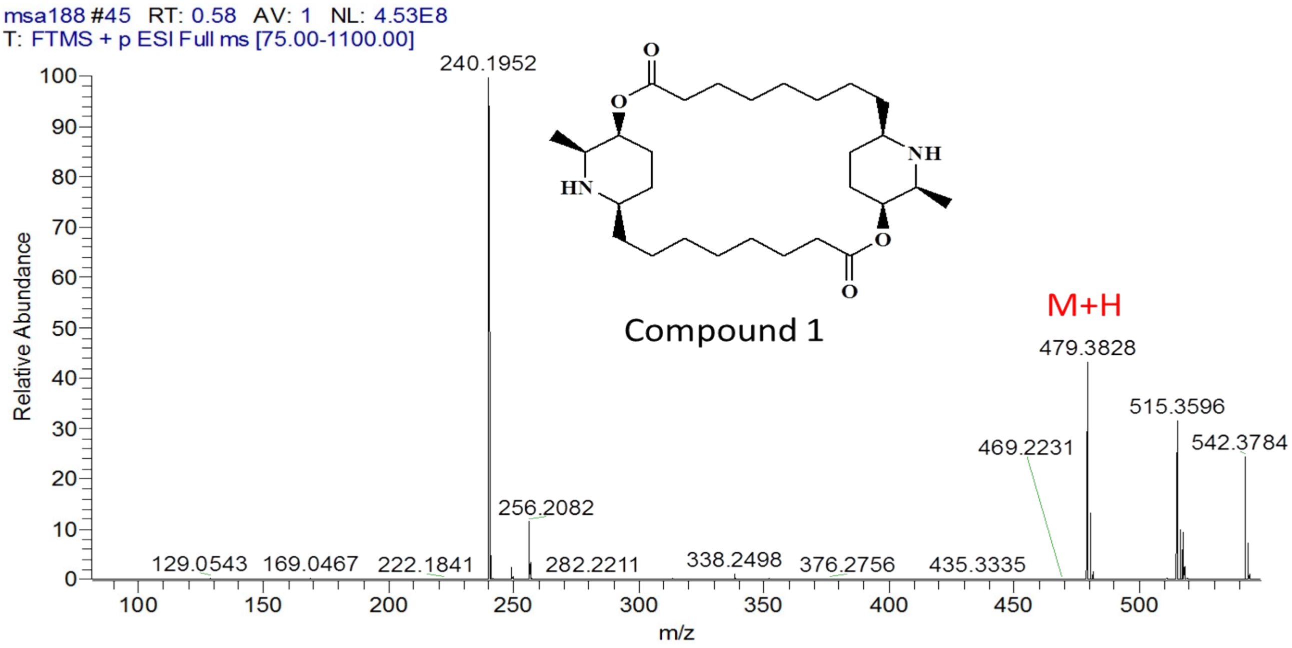

Fig. S6 Positive HR-ESI-MS spectrum of compound **1**.

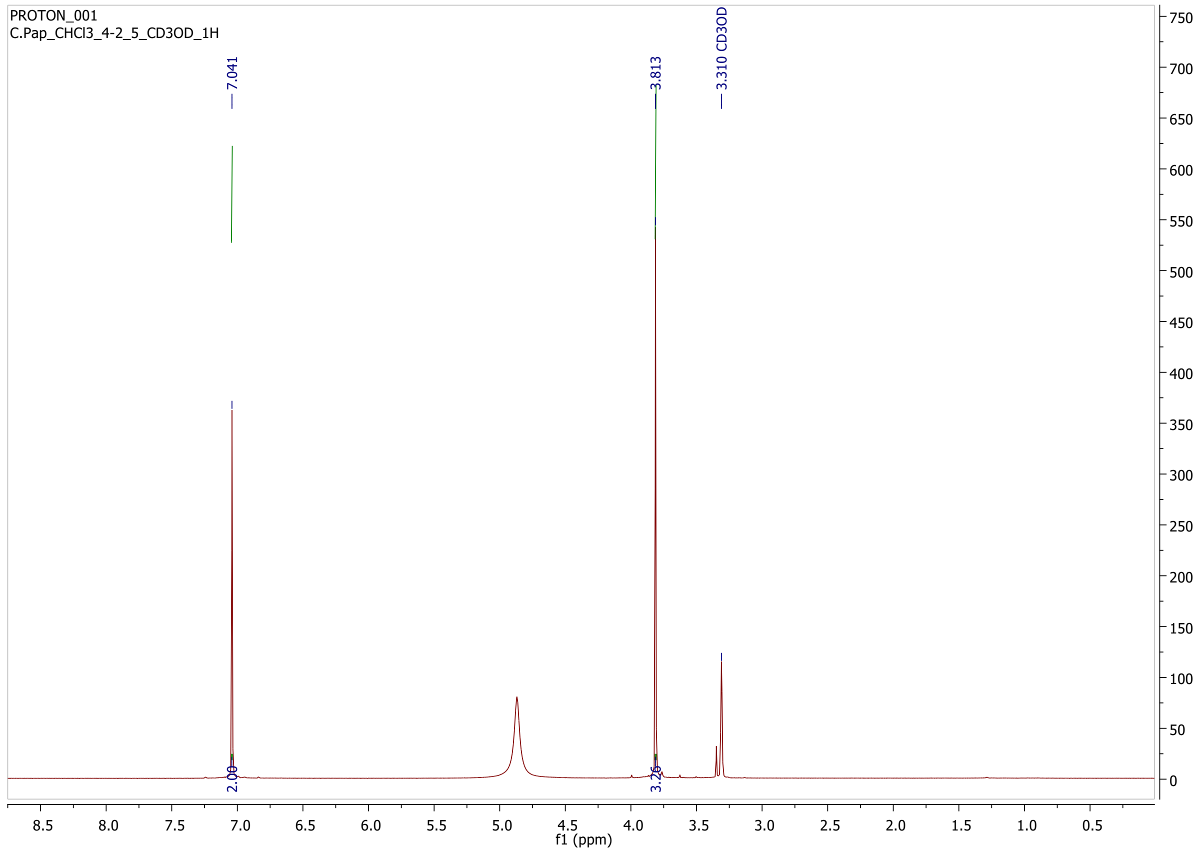

Fig. S7 ^{1}H-NMR spectrum of compound **2** (400 MHz, CD_3OD).

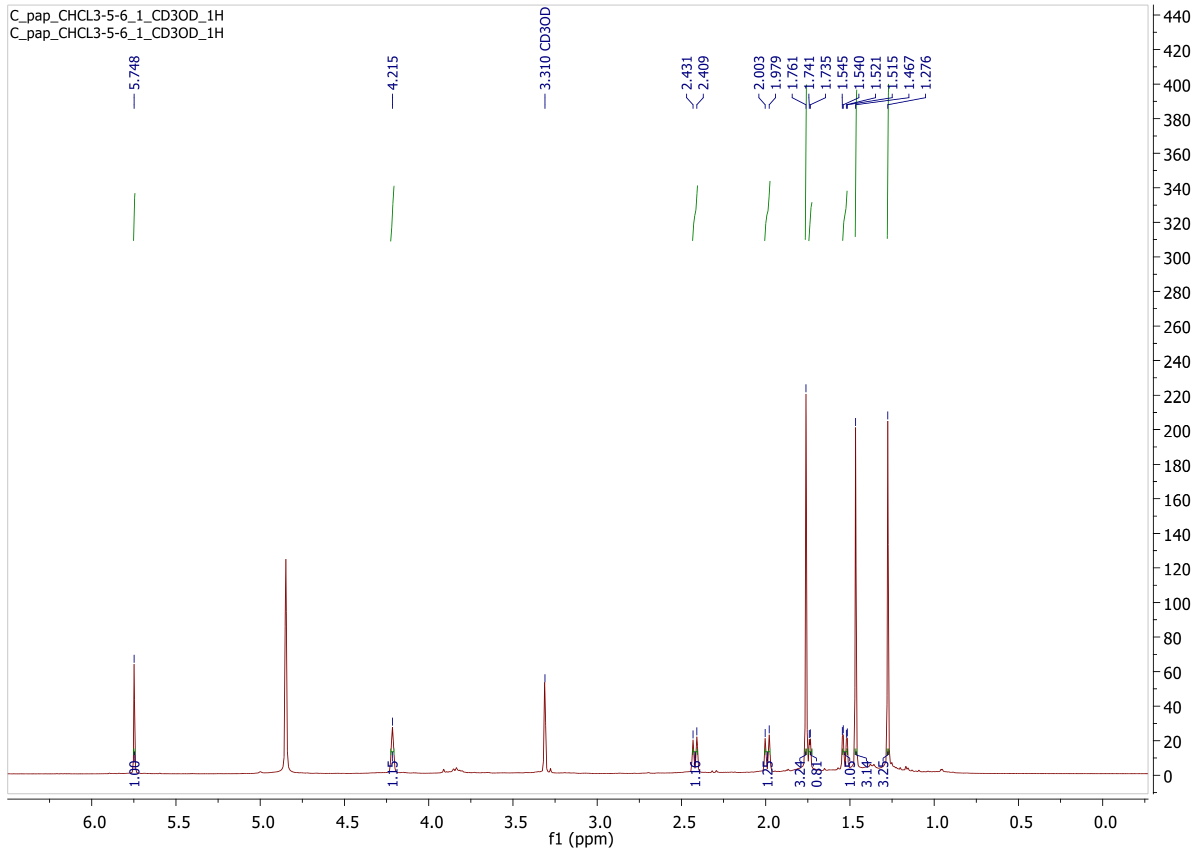

Fig. S8 ^{1}H-NMR spectrum of compound **3** (400 MHz, CD_3OD).

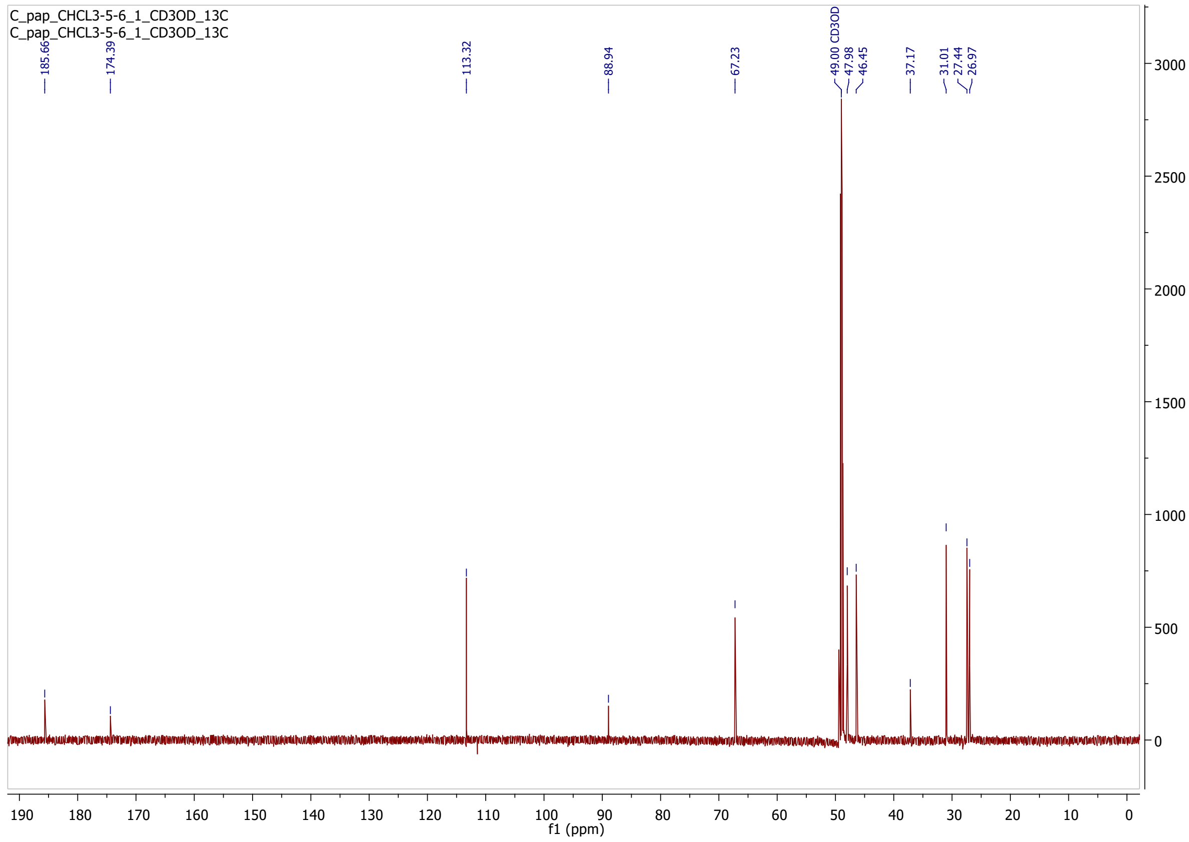

Fig. S9 ^{13}C-NMR spectrum of compound **3** (100 MHz, CD_3OD).

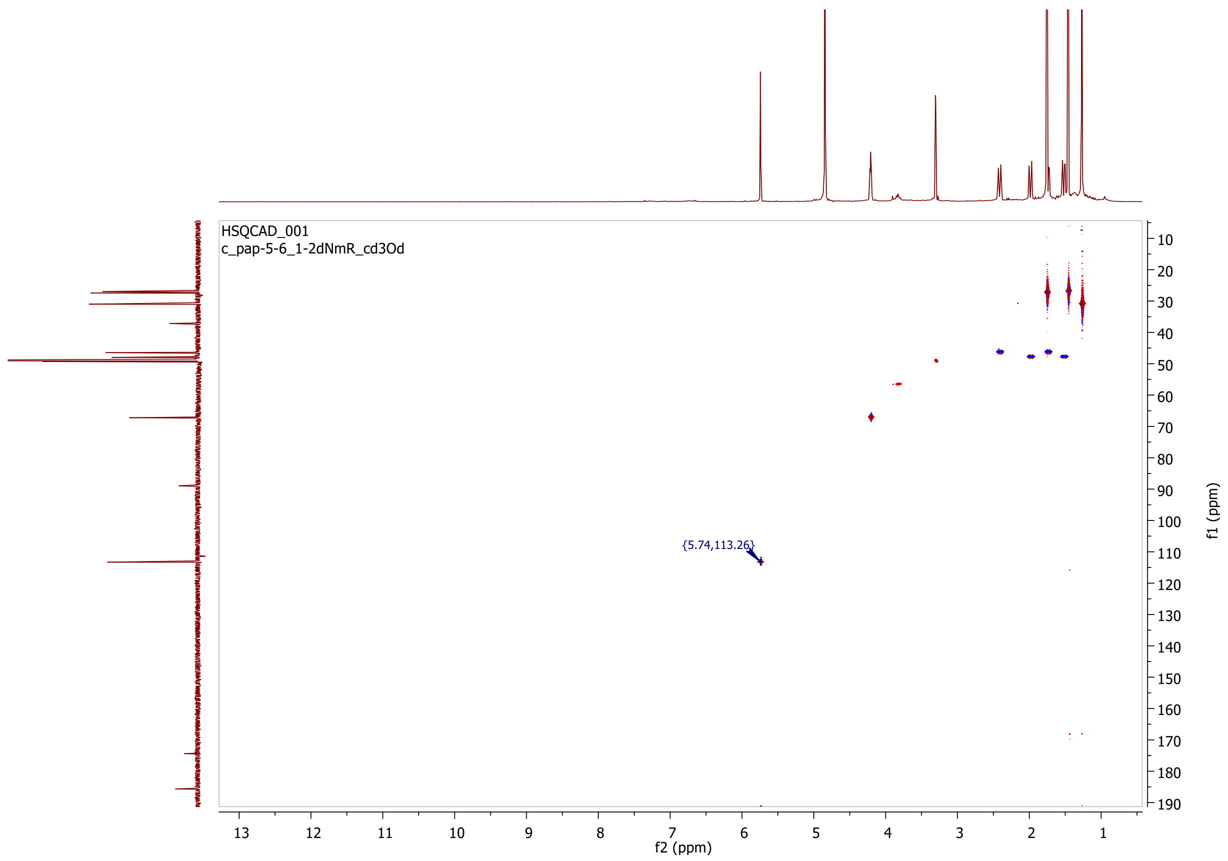

Fig. S10 HSQC spectrum of compound **3**.

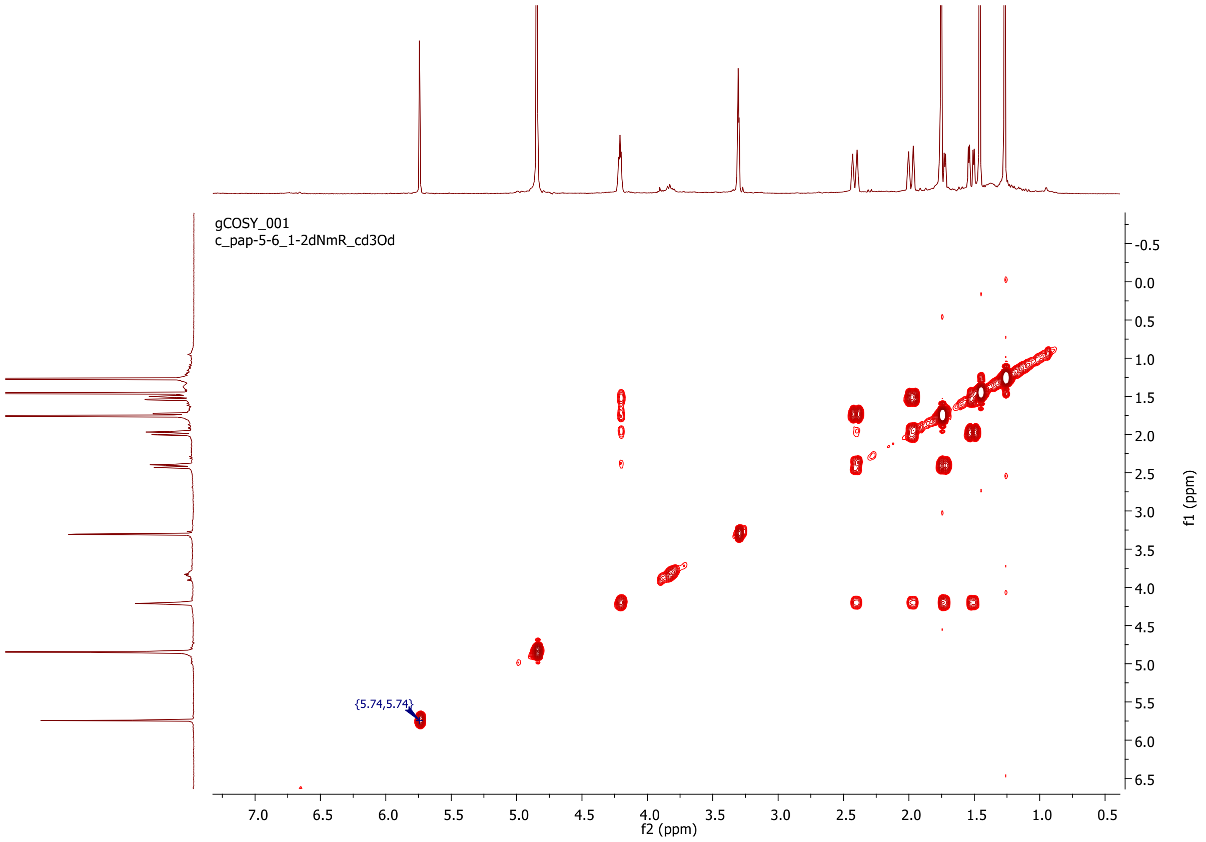

Fig. S11. ^{1}H-^{1}H COSY spectrum of compound **3**.

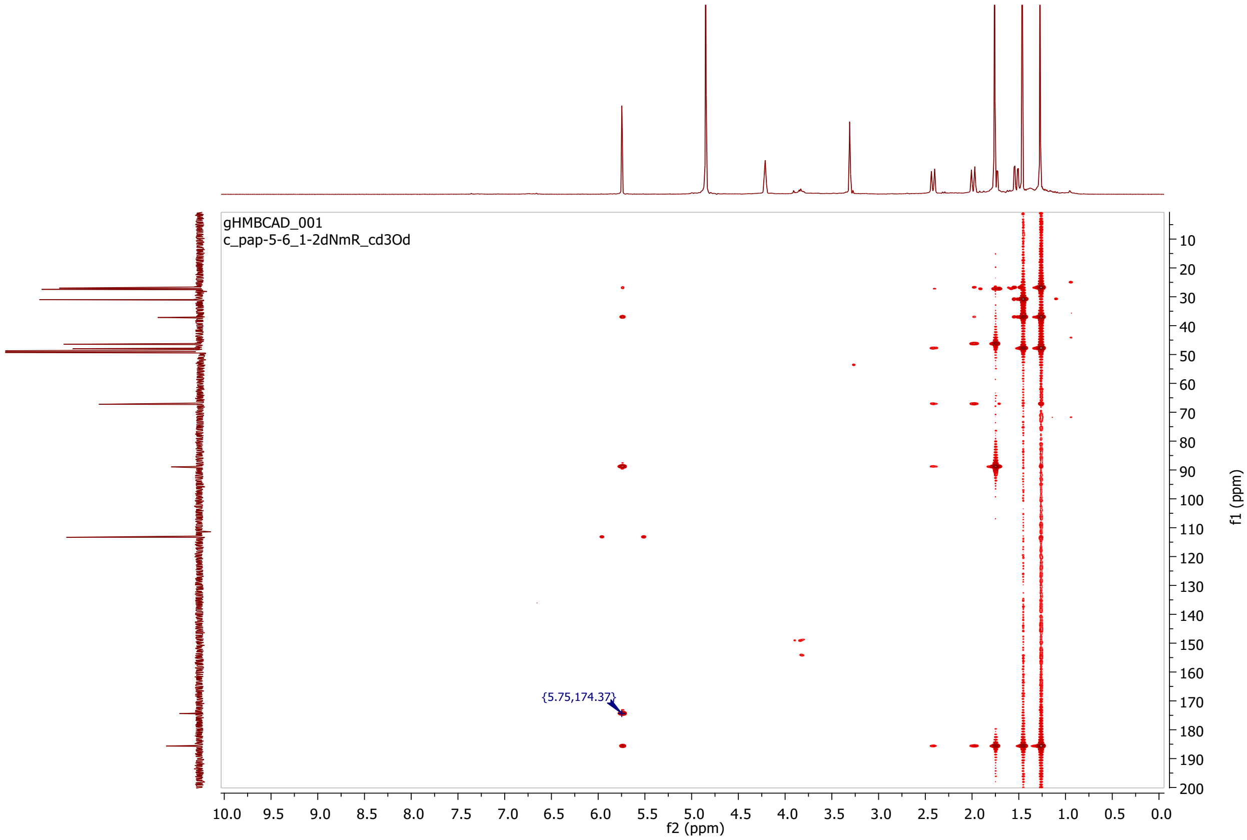

Fig. S12 HMBC spectrum of compound **3**.

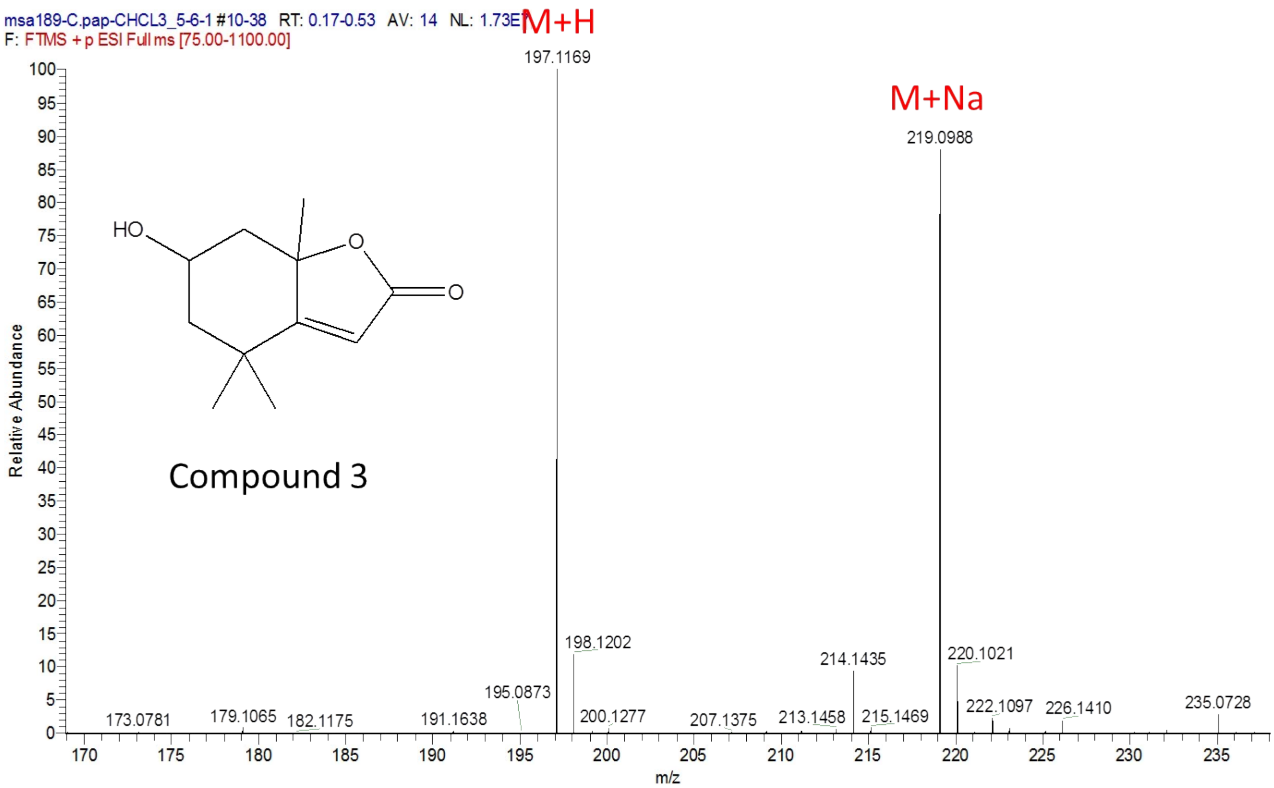

Fig. S13 Positive HR-ESI-MS spectrum of compound **3**.

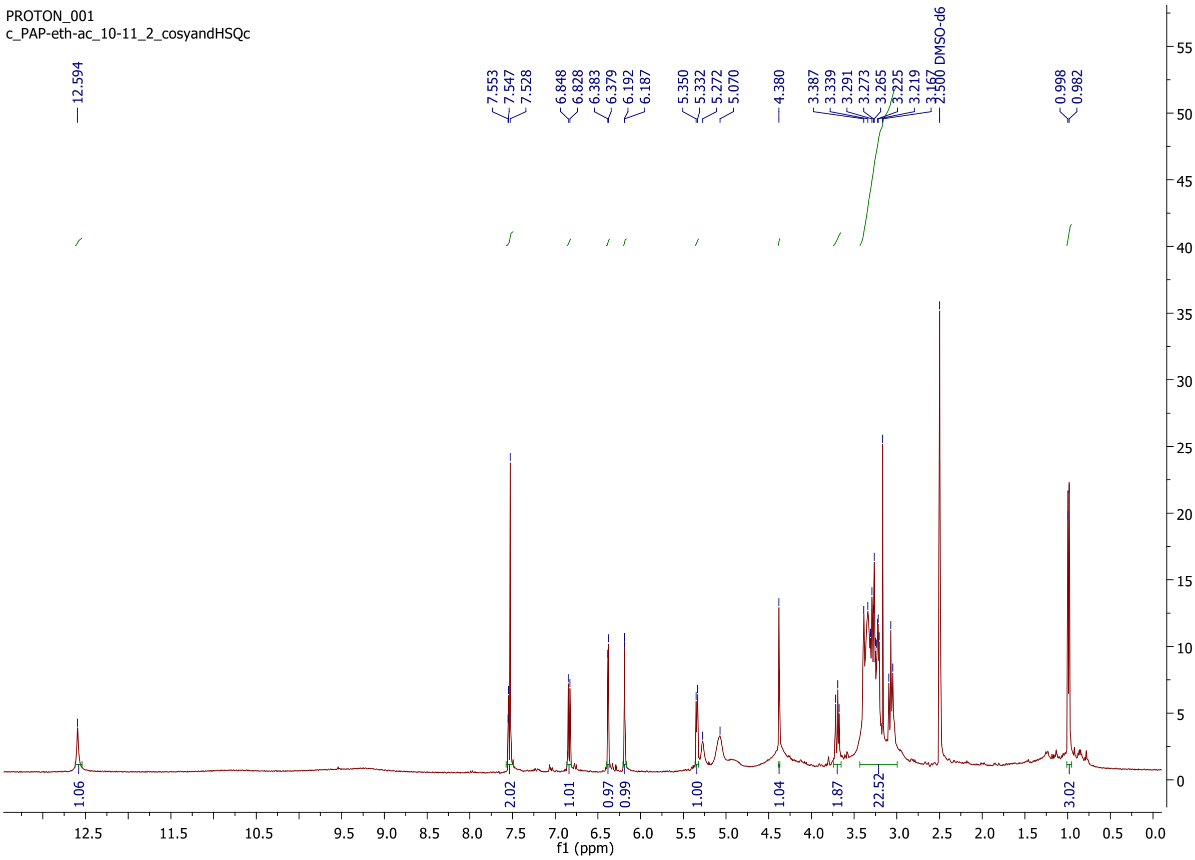

Fig. S14. ^{1}H-NMR spectrum of compound **4** (400 MHz, DMSO-d_6).

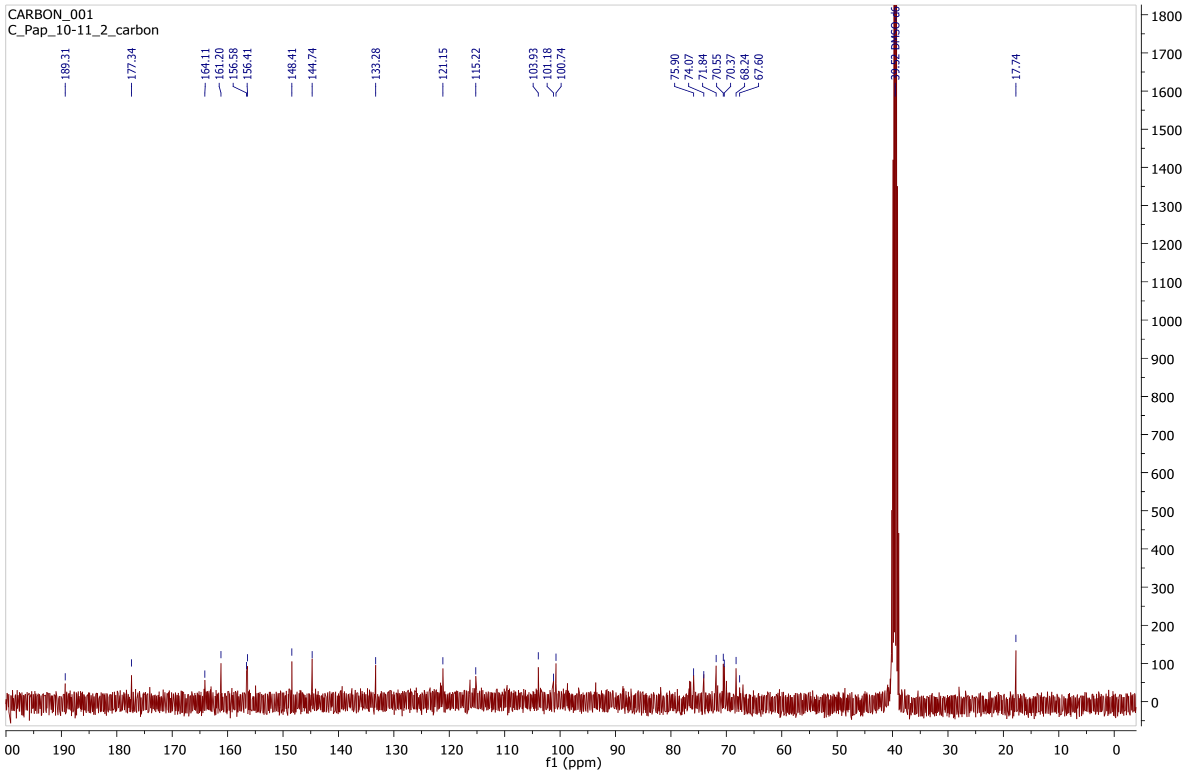

Fig. S15. ^{13}C-NMR spectrum of compound **4** (100 MHz, DMSO-d_6).

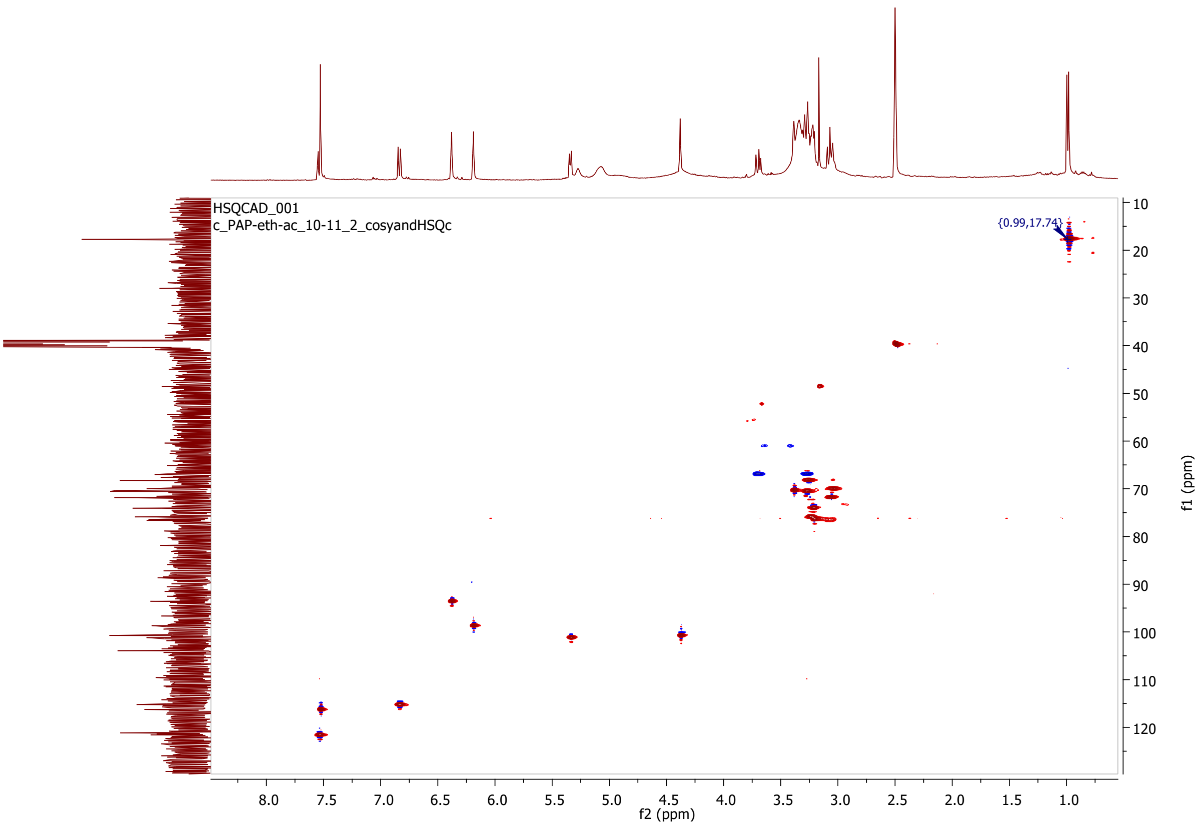

Fig. S16 HSQC spectrum of compound **4**.

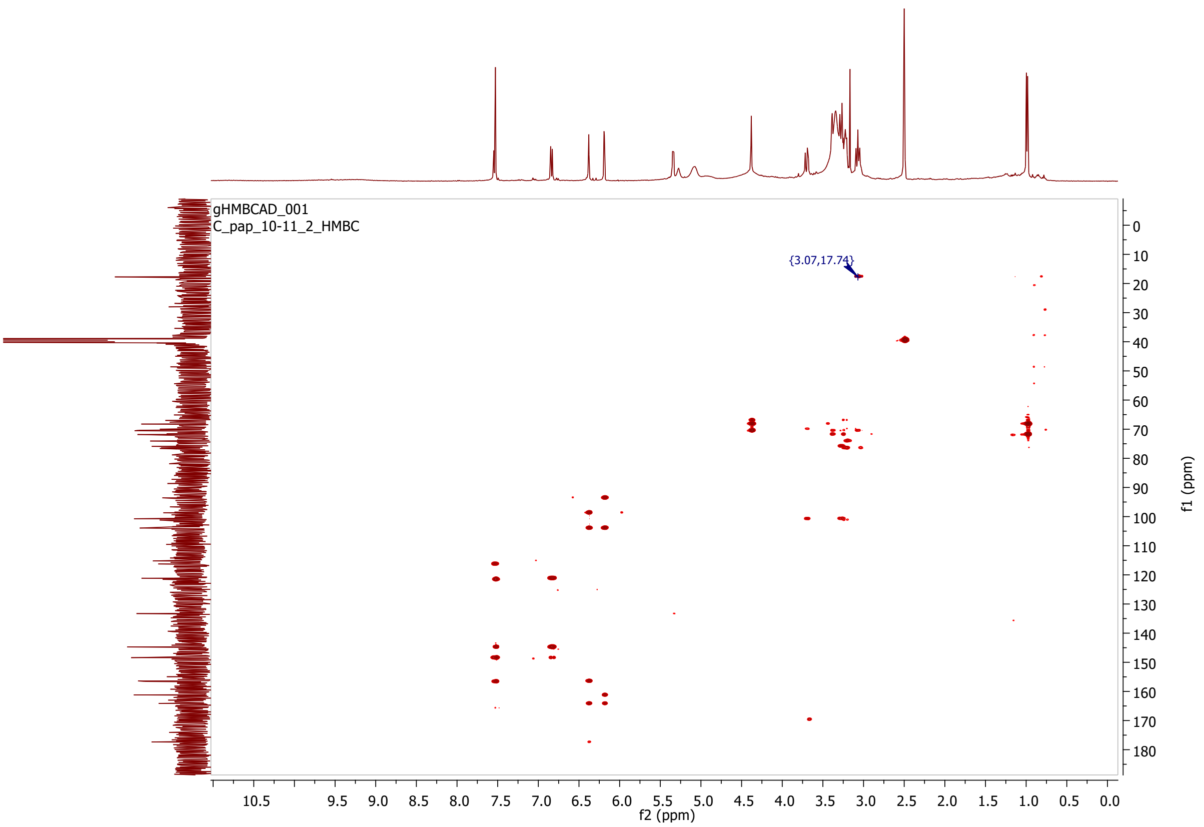

Fig. S17 HMBC spectrum of compound **4**.

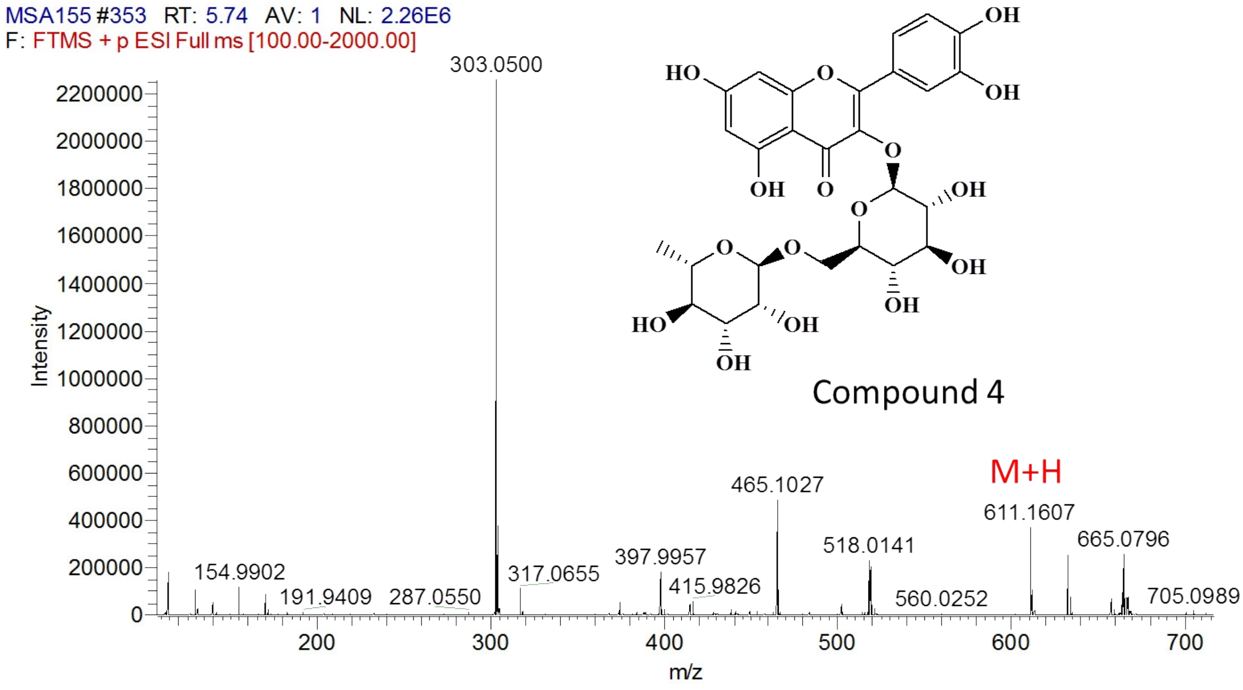

Fig. S18 Positive HR-ESI-MS spectrum of compound **4**.

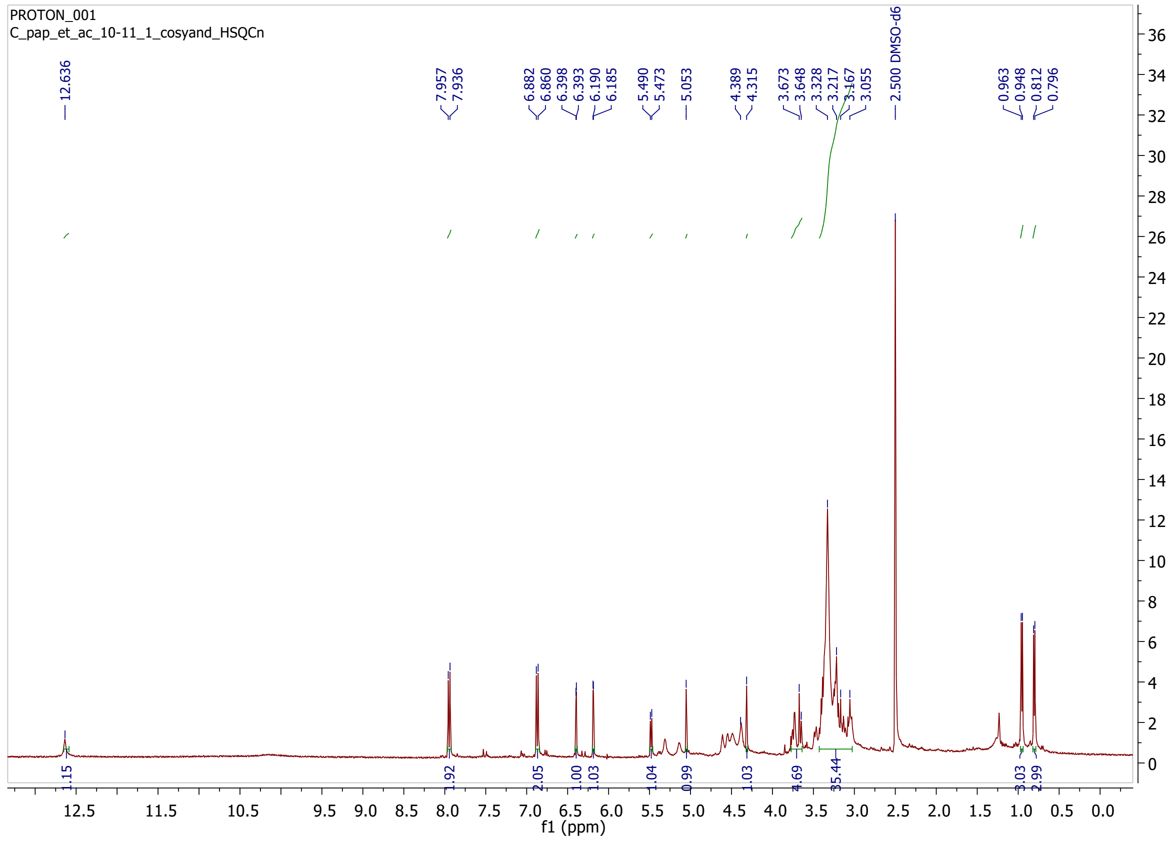

Fig. S19 ^{1}H-NMR spectrum of compound **5** (400 MHz, DMSO-d_6).

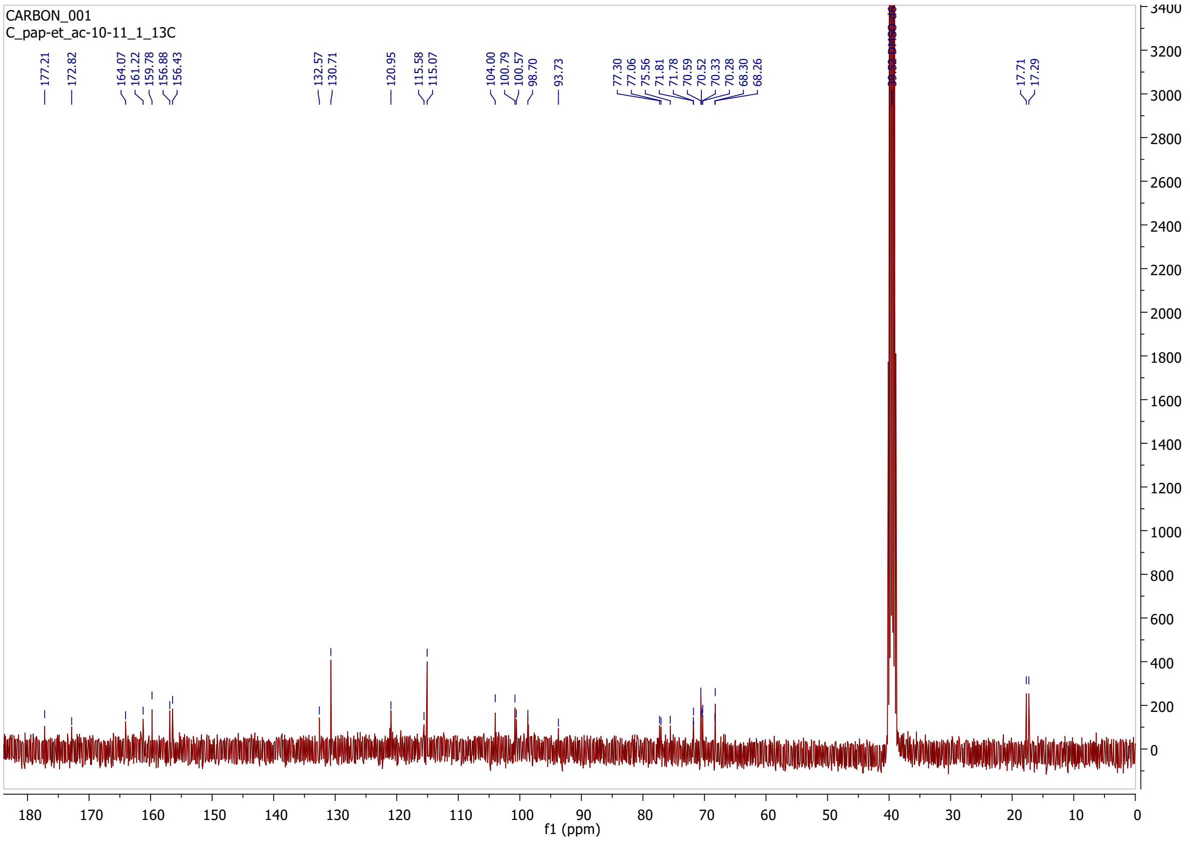

Fig. S20 ^{13}C-NMR spectrum of compound **5** (100 MHz, DMSO-d_6).

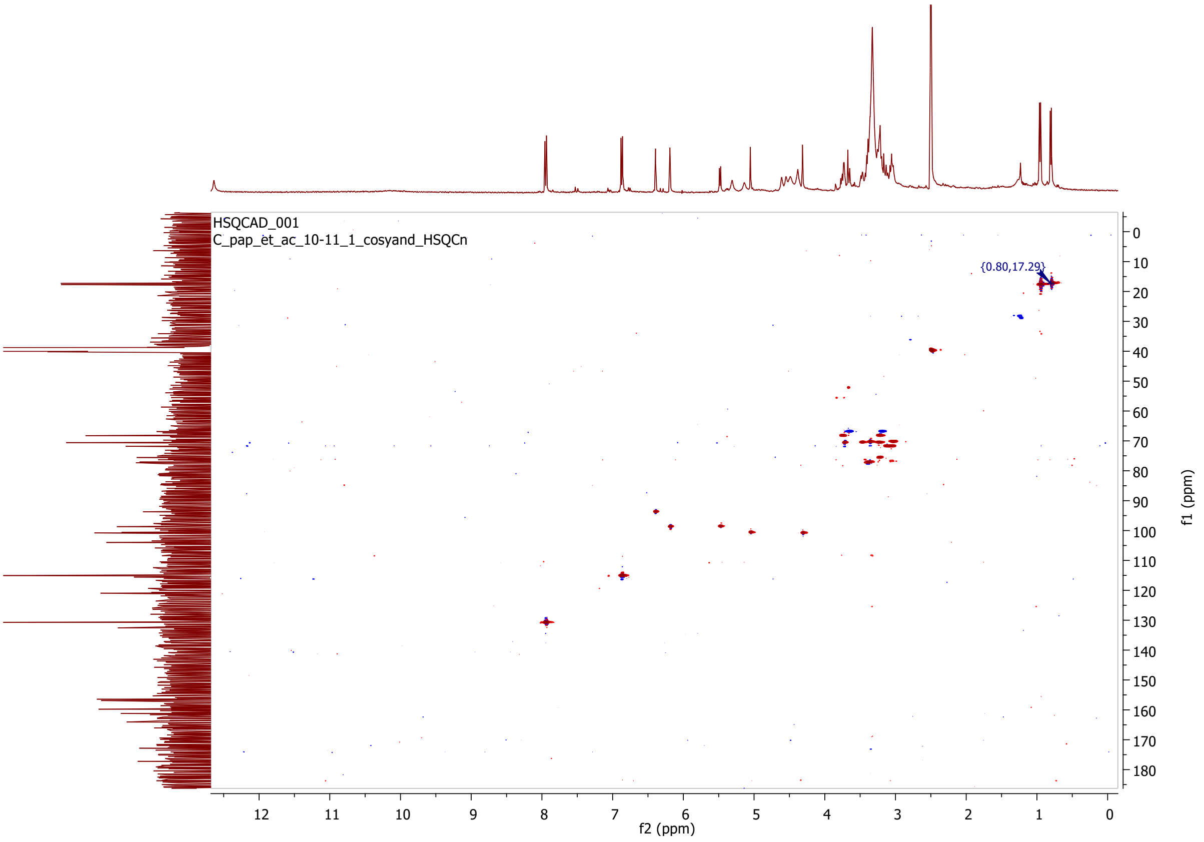

Fig. S21 HSQC spectrum of compound **5**.

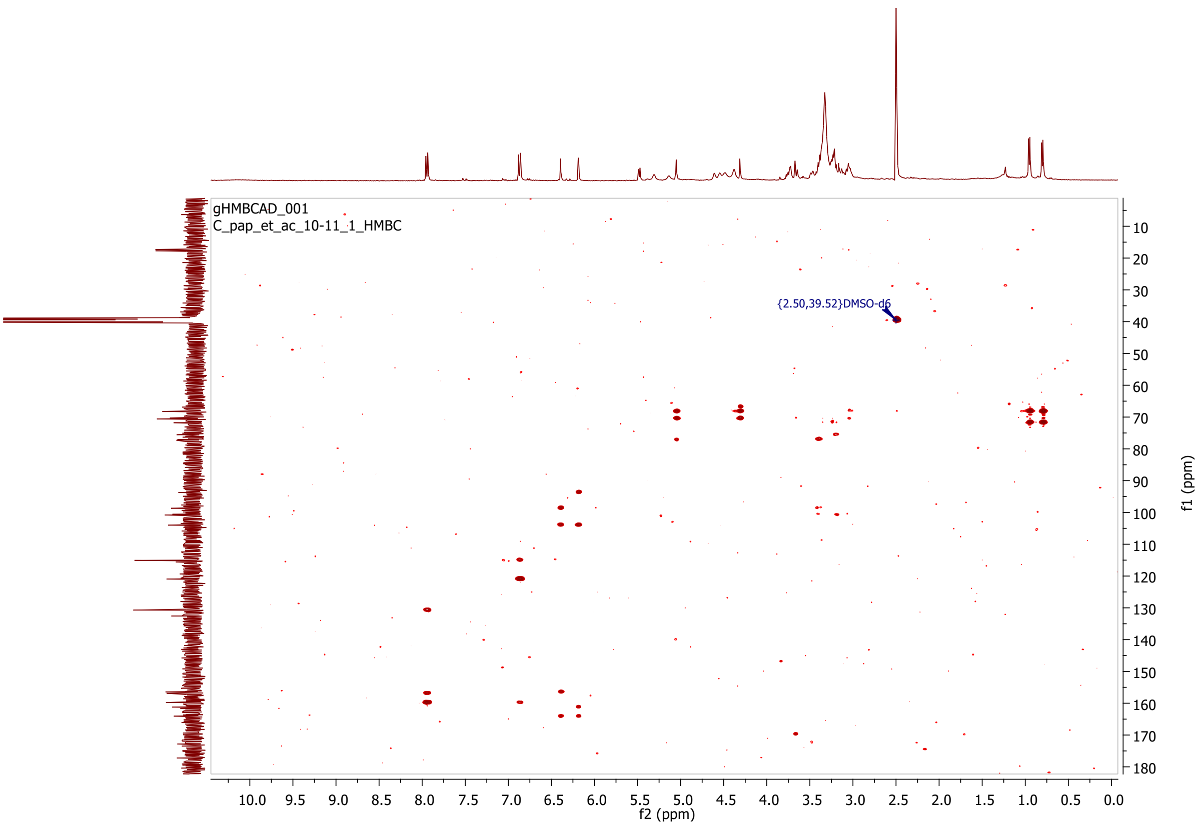

Fig. S22 HMBC spectrum of compound **5**.

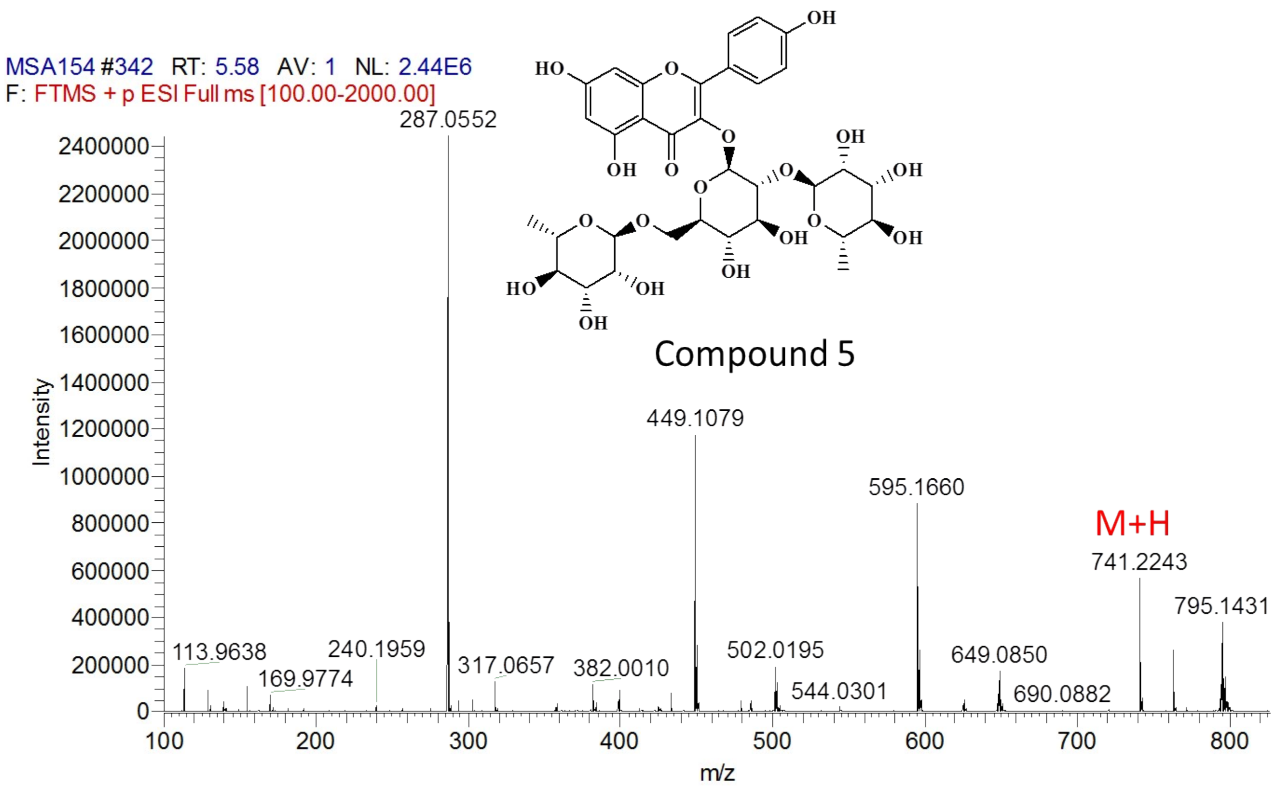

Fig. S23 Positive HR-ESI-MS spectrum of compound **5**.

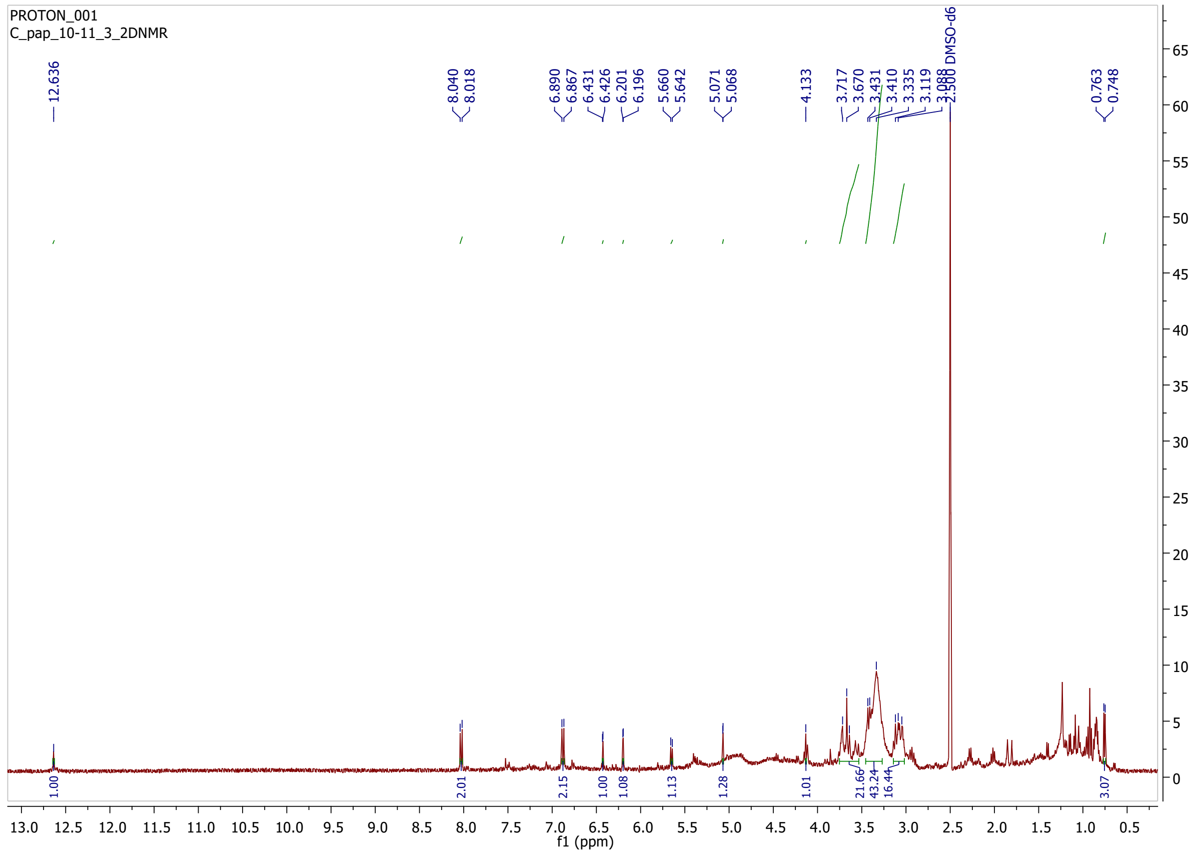

Fig. S24 ^{1}H-NMR spectrum of compound **6** (400 MHz, DMSO-d_6).

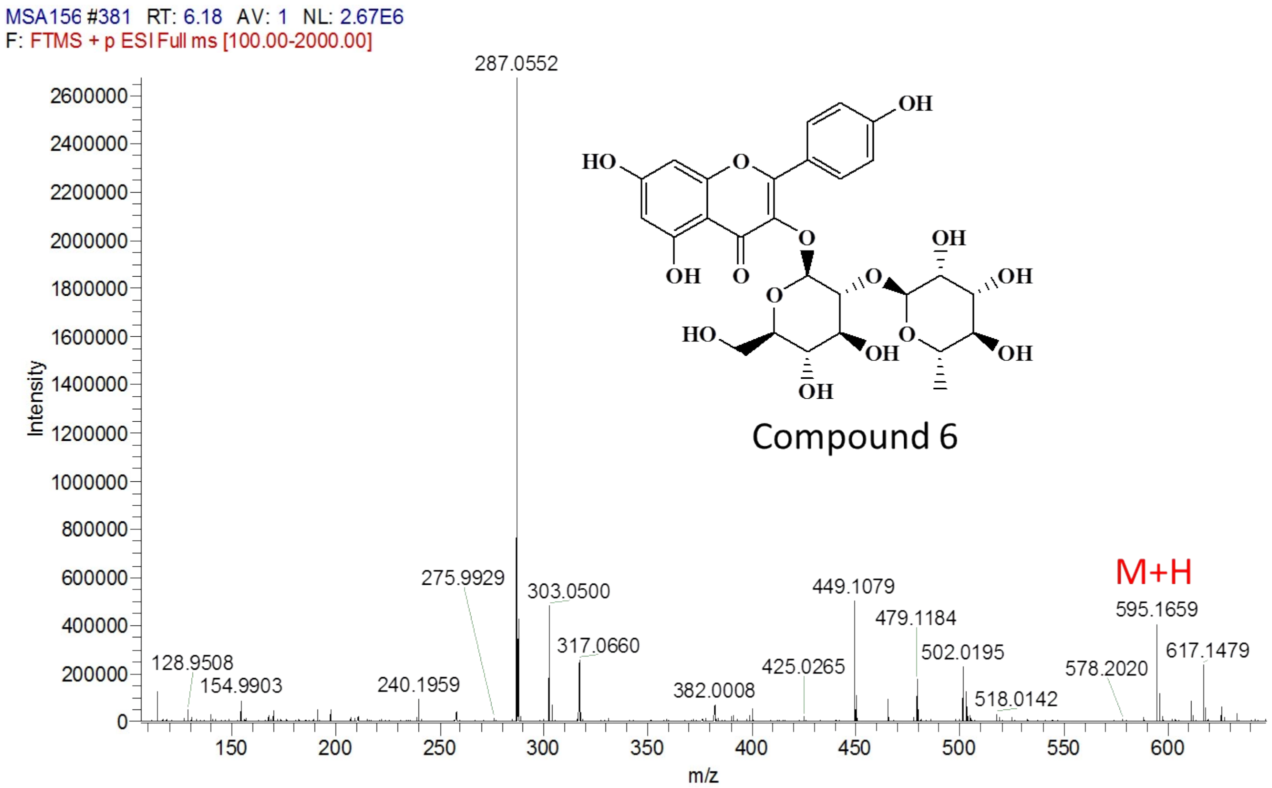

Fig. S25 Positive HR-ESI-MS spectrum of compound **6**.

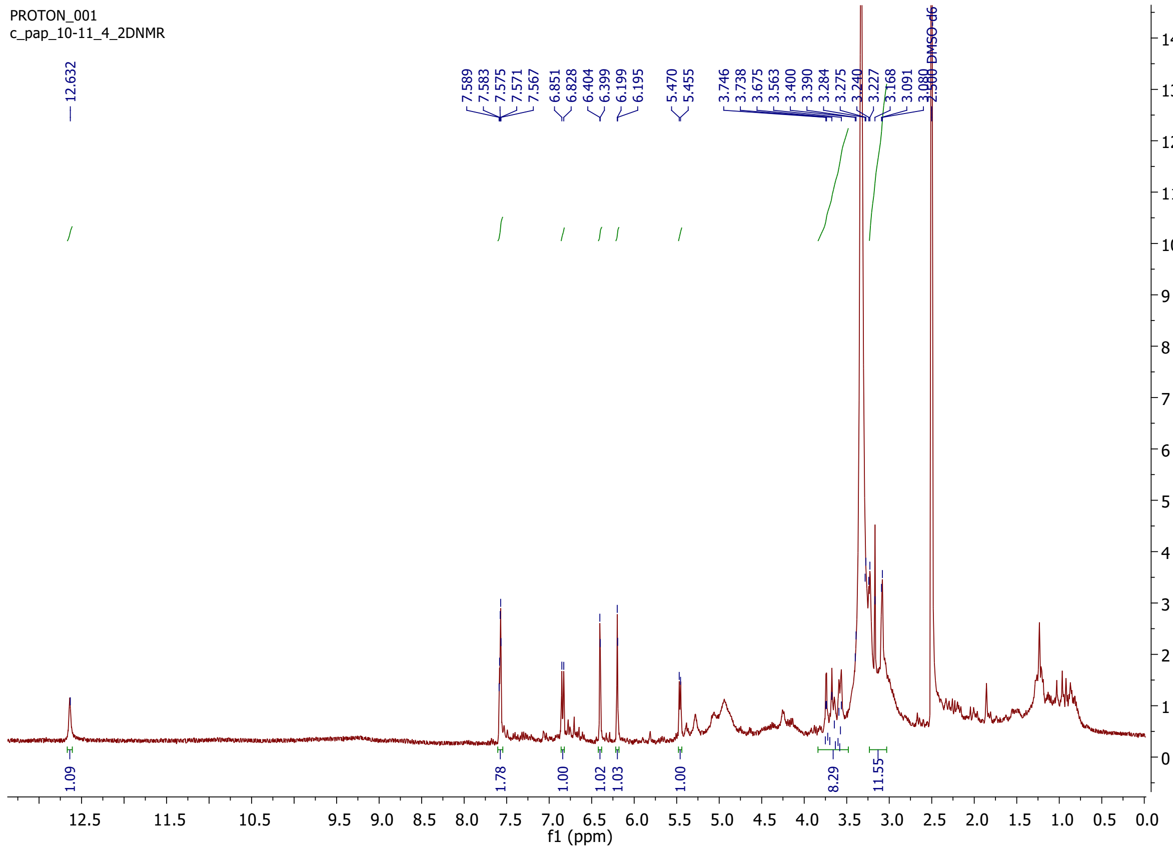

Fig. S26 ^{1}H-NMR spectrum of compound **7** (400 MHz, DMSO-d_6).

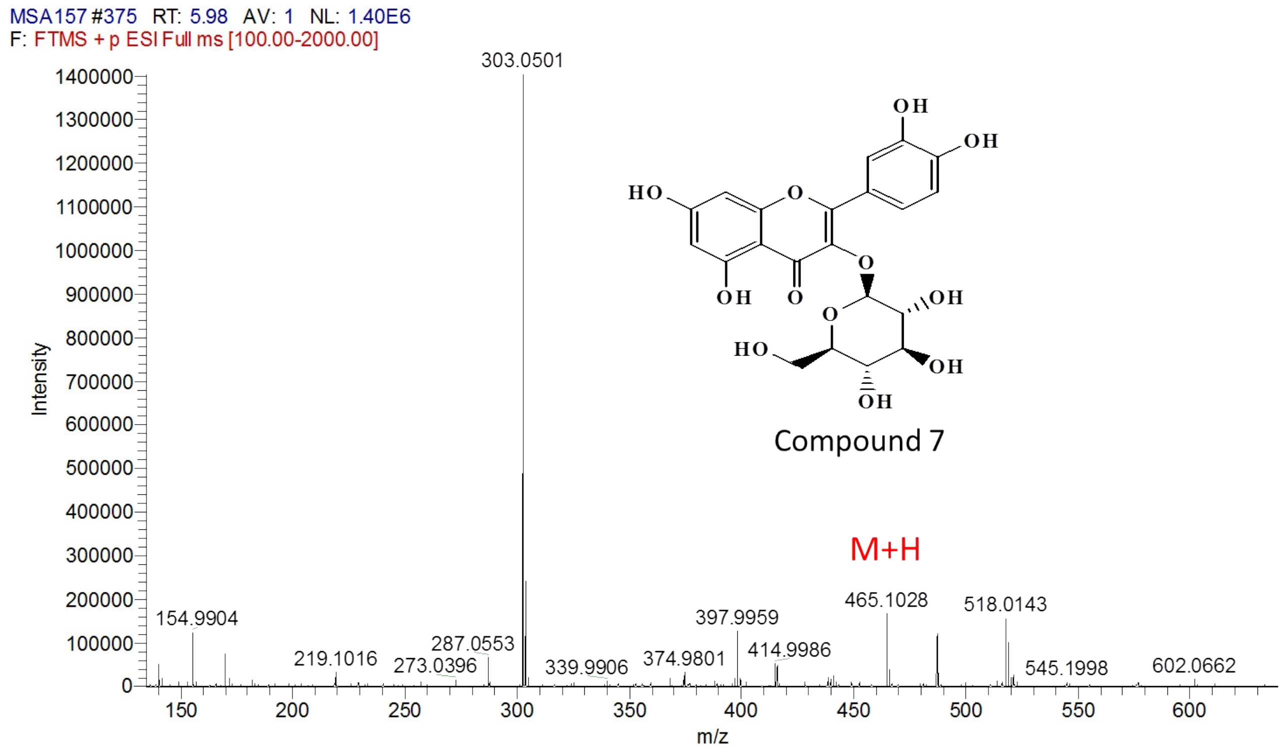

Fig. S27 Positive HR-ESI-MS spectrum of compound **7**.

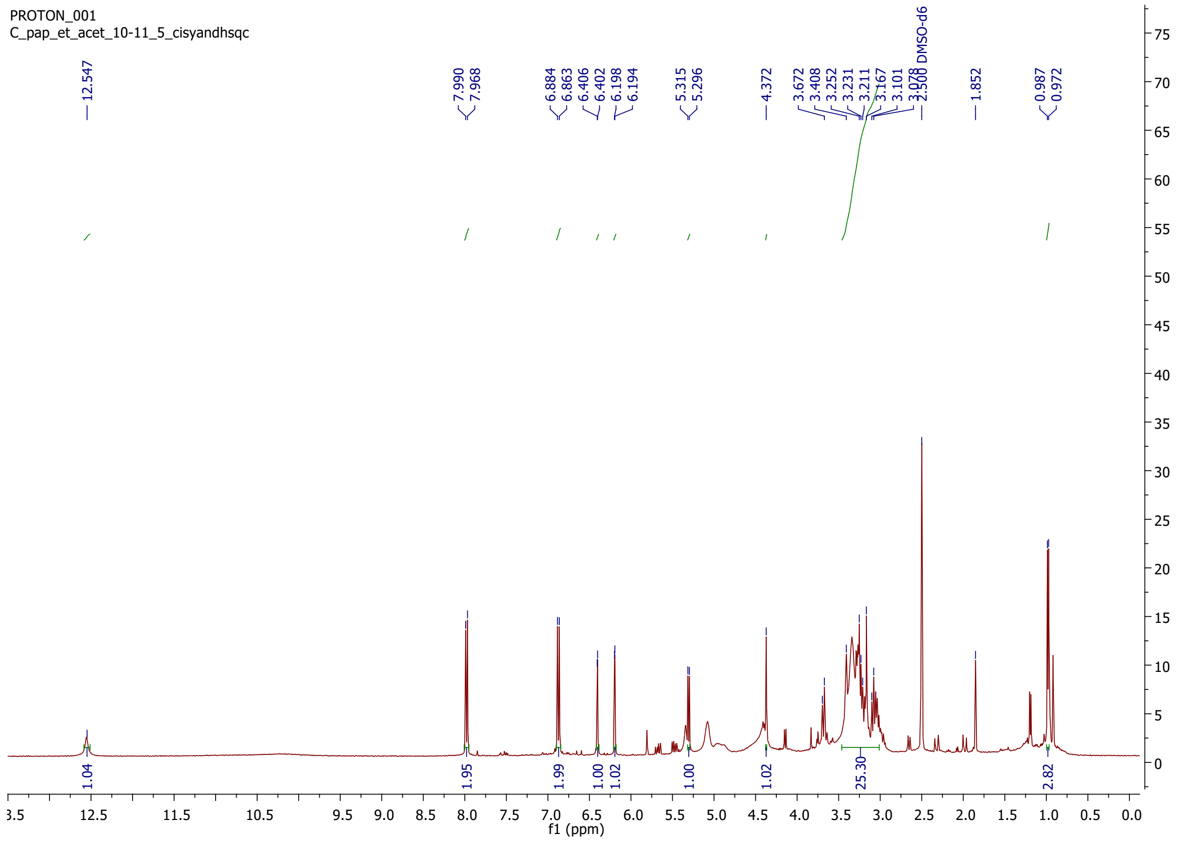

Fig. S28 ^{1}H-NMR spectrum of compound **8** (400 MHz, DMSO-d_6).

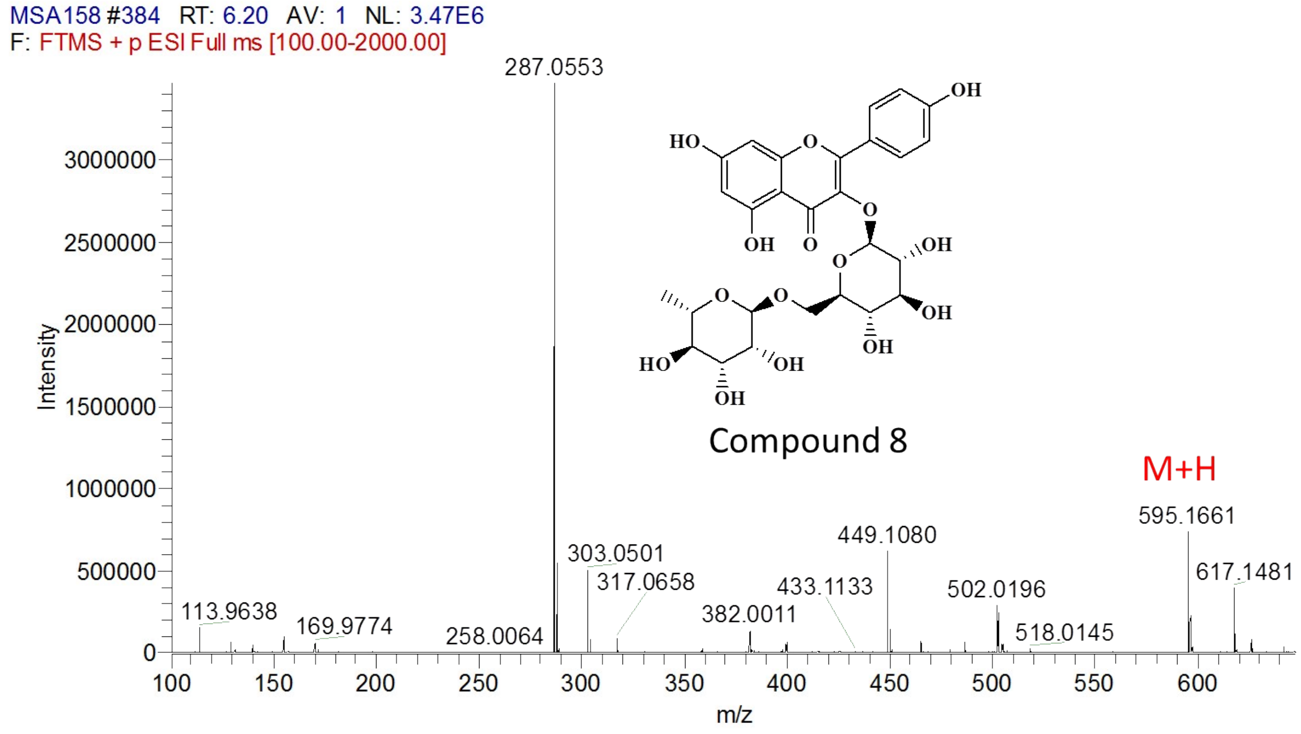

Fig. S29 Positive HR-ESI-MS spectrum of compound **8**.

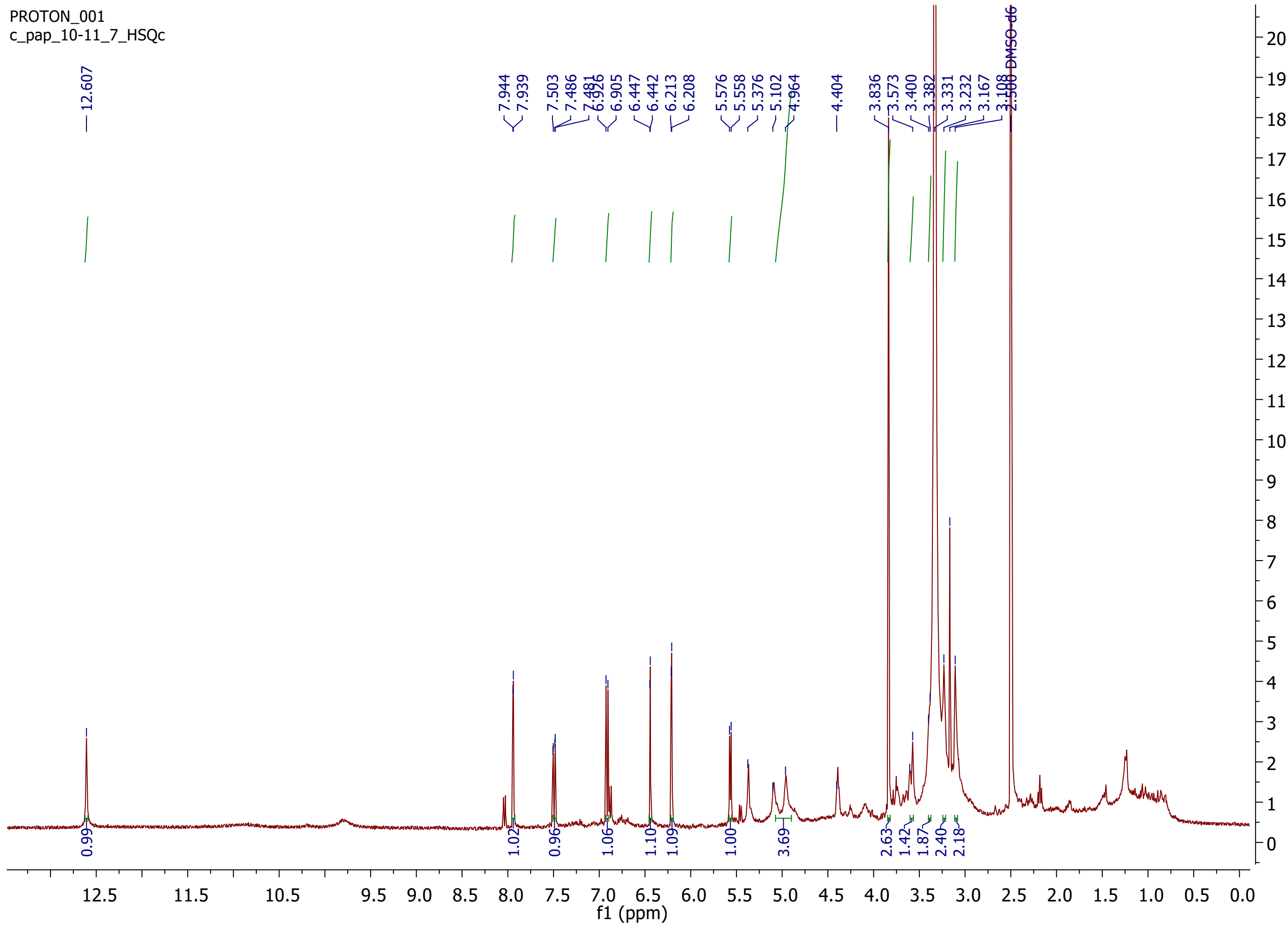

Fig. S30 ^{1}H-NMR spectrum of compound **9** (400 MHz, DMSO-d_6).

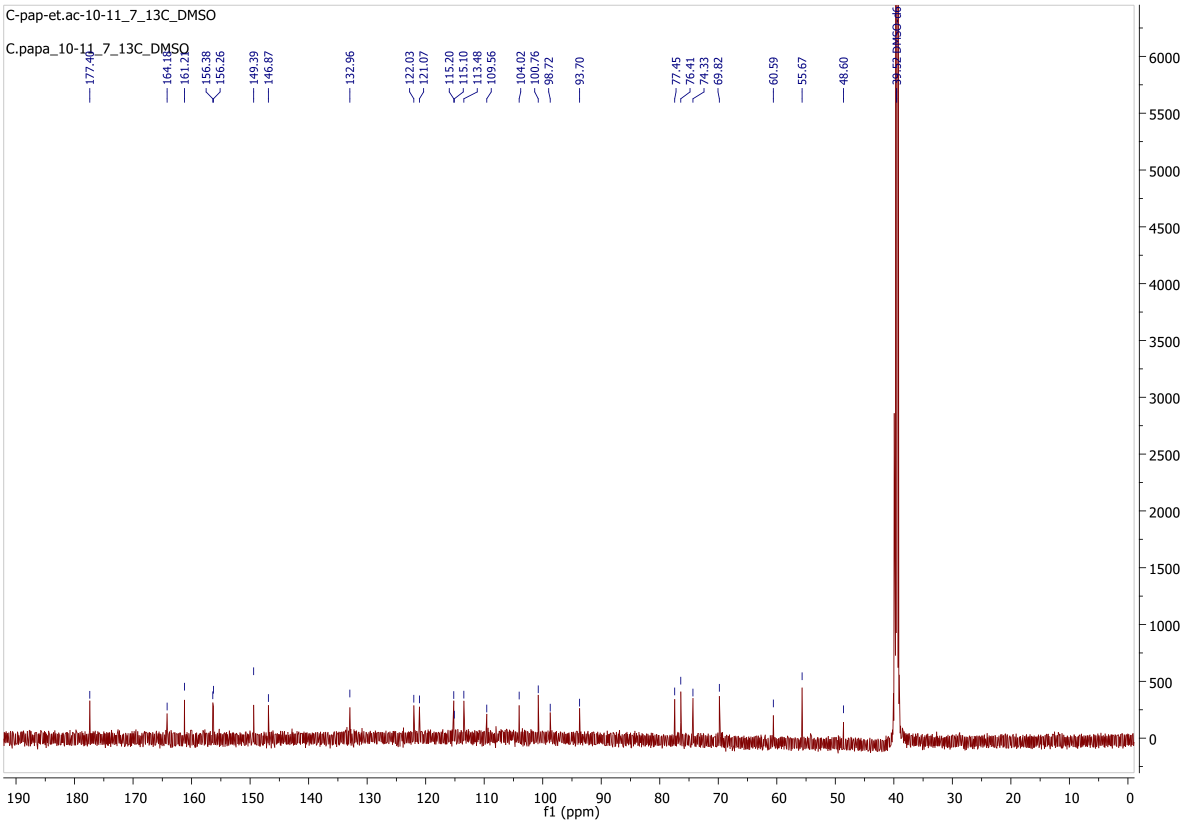

Fig. S31 ^{13}C-NMR spectrum of compound **9** (100 MHz, DMSO-d_6).

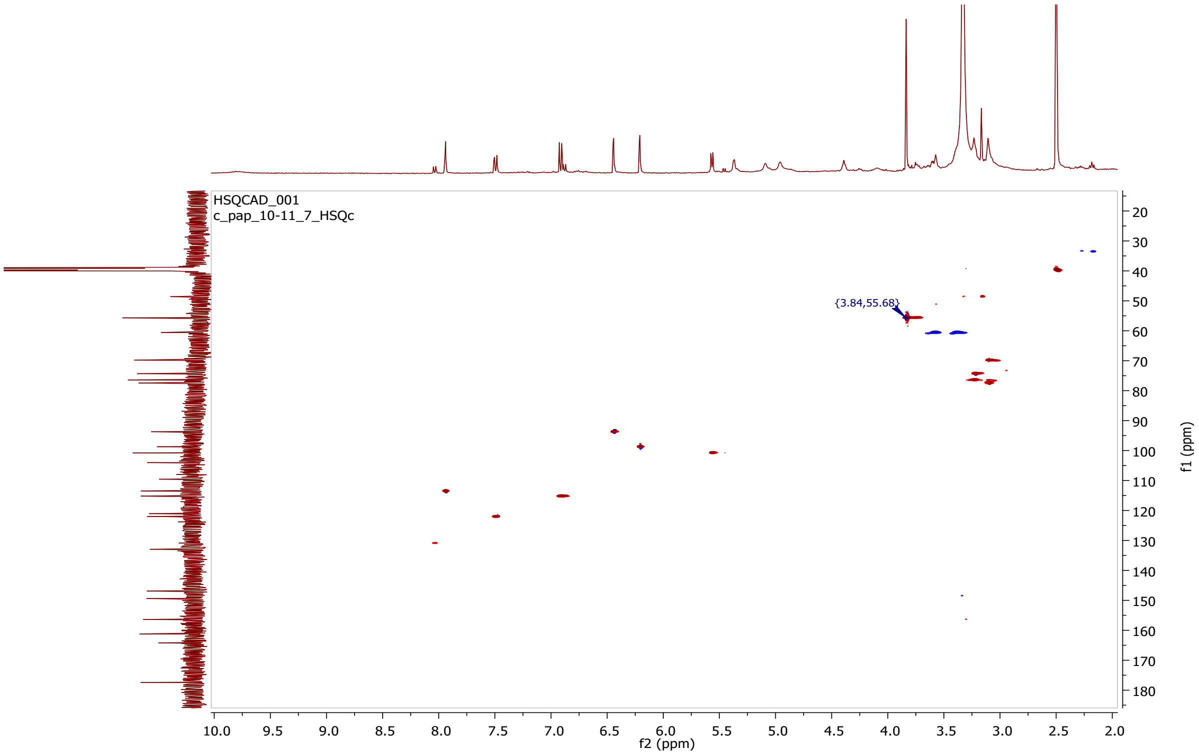

Fig. S32 HSQC spectrum of compound **9**.

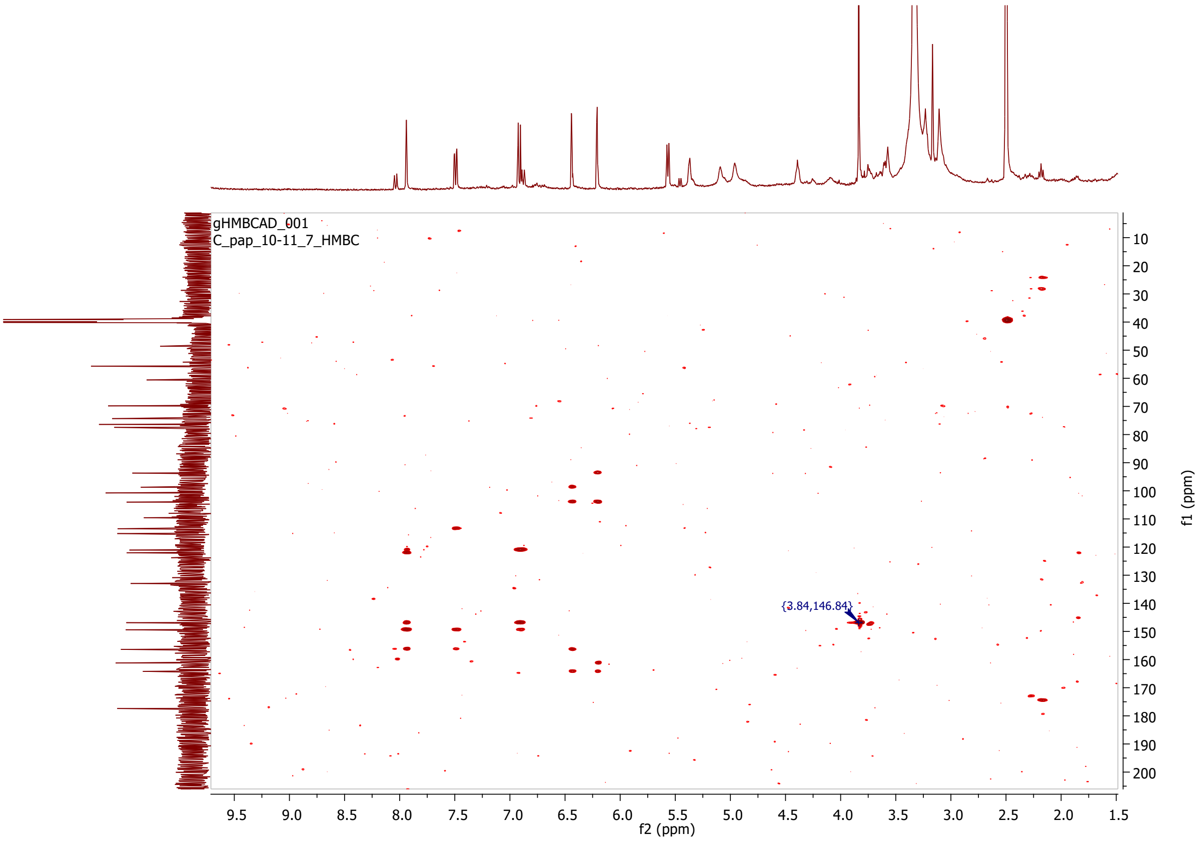

Fig. S33 HMBC spectrum of compound **9**.

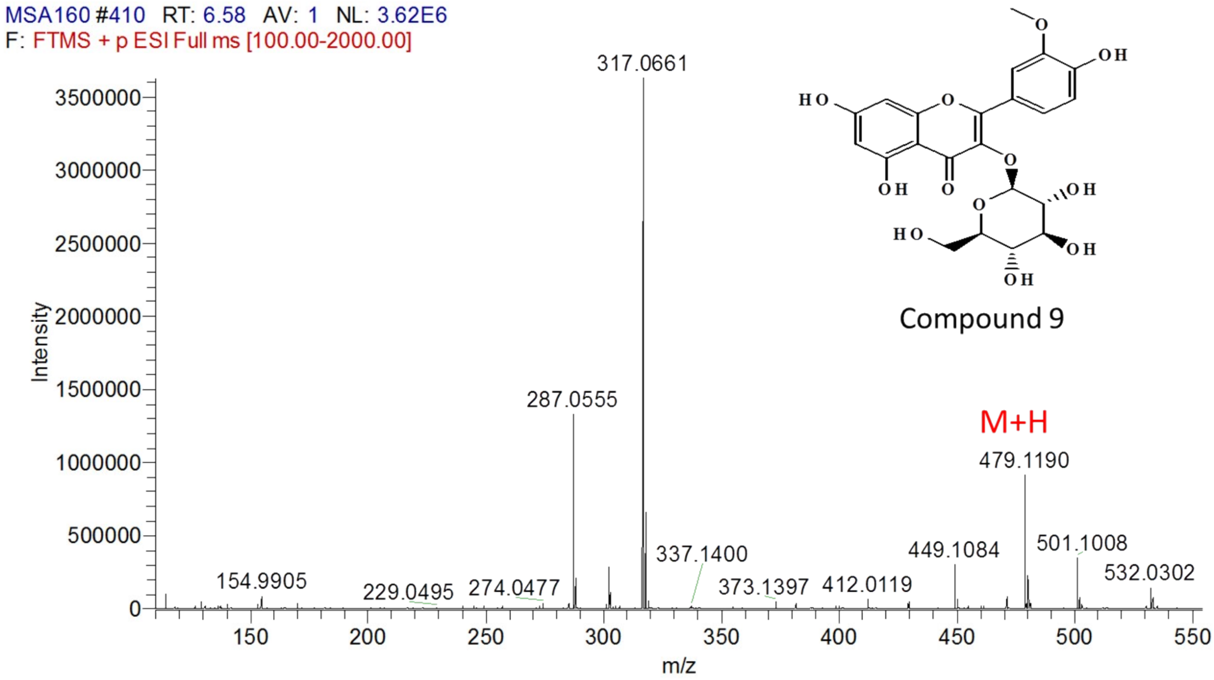

Fig. S34 Positive HR-ESI-MS spectrum of compound **9**.
